# Targeting ferroptosis in rotenone-induced Parkinson’s disease: from mechanistic insights to pharmacological interventions

**DOI:** 10.3389/fphar.2026.1976646

**Published:** 2026-10-28

**Authors:** Hongquan Wang, Zhiji Wang, Qiang Li, Shuang Wu, Yumin Wang

**Affiliations:** 1 Department of Geriatrics, Aerospace Center Hospital, Peking University Aerospace School of Clinical Medicine, Beijing, China; 2 Aerospace Medical Center, Aerospace Center Hospital, Beijing, China; 3 GENERTEC Key Laboratory of Aerospace Medical Research and Transformation, Beijing, China; 4 Department of Neurology, Hohhot First Hospital, Fifth Clinical Medical College, Inner Mongolia Medical University, Hohhot, China; 5 Department of Neurology, The Affiliated Hospital of Chifeng University, Chifeng, China; 6 Department of Neurology, Zhongnan Hospital of Wuhan University, Wuhan, China; 7 Hubei Provincial Clinical Research Center for Dementia and Cognitive Impairment, Wuhan, Hubei, China

**Keywords:** ferroptosis, ferroptosis inhibitor, neurodegenerative disorder, Parkinson’s disease, rotenone

## Abstract

Parkinson’s disease (PD) is a progressive neurodegenerative disorder characterized by the selective loss of dopaminergic neurons in the substantia nigra, leading to disabling motor and non-motor symptoms. Ferroptosis, an iron-dependent oxidative cell death driven by lethal lipid peroxidation, has emerged in preclinical models as a candidate pathogenic mechanism in PD, although its causal centrality in human PD remains to be established. Rotenone, a mitochondrial complex I inhibitor widely used to model PD, recapitulates key clinical and pathological features of the disease. Accumulating evidence demonstrates that rotenone induces ferroptosis through a coordinated network of iron dyshomeostasis, inactivation of the System Xc^−^–GPX4 antioxidant axis, dysregulated lipid metabolism, mitochondrial dysfunction, ferritinophagy-mediated iron release, neuroinflammation, and SIRT1–Nrf2 pathway suppression. Importantly, pharmacological targeting of these ferroptotic nodes—with agents such as Fer-1, iron chelators, Nrf2 activators, and modulators of ferritinophagy—effectively attenuates rotenone-induced neurodegeneration and restores motor function in preclinical models. This review systematically dissects the molecular circuitry of ferroptosis in rotenone -based PD models and evaluates the therapeutic potential of ferroptosis inhibitors, aiming to inform the development of disease-modifying strategies for PD.

## 1 Introduction

Parkinson’s disease (PD) is the second most common neurodegenerative disorder, characterized by the progressive loss of dopaminergic neurons in the substantia nigra pars compacta and the accumulation of α-synuclein-containing Lewy bodies (Kalia and Lang, 2015; Poewe et al., 2017). While the etiology of PD remains incompletely understood, both genetic and environmental factors have been implicated in its pathogenesis. Among environmental toxins, rotenone—a naturally occurring pesticide and potent mitochondrial complex I inhibitor—has been extensively used to model PD in experimental systems, as it reproduces many of the cardinal pathological and behavioral features of the human disease, including selective nigrostriatal dopaminergic degeneration, α-synuclein aggregation, and motor deficits (Betarbet et al., 2000; Cannon et al., 2009).

Regulated cell death (RCD) encompasses a diverse array of molecularly distinct pathways that play critical roles in physiological homeostasis and the pathogenesis of numerous diseases. Among these, ferroptosis has emerged in preclinical studies as a contributory mechanism in rotenone-induced neurotoxicity, but its causal role in human PD is not yet established. Ferroptosis is an iron-dependent, oxidative form of non-apoptotic cell death driven by the peroxidation of polyunsaturated fatty acid (PUFA)-containing membrane phospholipids (Yang and Stockwell, 2008; Dixon et al., 2012; Stockwell, 2022; Wang et al., 2023a). It is governed by intricate metabolic and antioxidant networks, including the System Xc^−^–Glutathione peroxidase 4 (GPX4) axis, which serves as the primary defense against lipid peroxidation (Yang et al., 2014; Kagan et al., 2017), and parallel surveillance systems such as the CoQ10–FSP1 and DHODH pathways (Bersuker et al., 2019; Doll et al., 2019; Mao et al., 2021; Mishima et al., 2022). The sensitivity of cells to ferroptosis is largely determined by the abundance of PUFA-containing lipids, which serve as substrates for lipid peroxidation, and by the activity of key enzymes such as acyl-CoA synthetase long-chain family member 4 (ACSL4) and lysophosphatidylcholine acyltransferase 3 (LPCAT3) that promote PUFA incorporation into membranes (Doll et al., 2017; Liang et al., 2023).

Rotenone exposure induces ferroptosis through multiple interconnected mechanisms: disruption of iron homeostasis leading to iron accumulation and Fenton chemistry-driven lipid peroxidation; inactivation of the System Xc^−^–GPX4 antioxidant axis; dysregulation of lipid metabolism with accumulation of pro-ferroptotic signals; mitochondrial dysfunction that amplifies oxidative stress; and ferritinophagy-mediated iron release that further propagates ferroptotic death (Hu et al., 2023; Ma et al., 2024; Sun et al., 2024; Liu et al., 2025). However, iron accumulation, oxidative stress and lipid peroxidation are not equivalent to ferroptosis; ferroptosis-specific evidence requires GPX4/ACSL4-dependent lipid peroxidation, oxidized PUFA-PE signals, and rescue by Fer-1/Lipo-1 or DFO rather than by general antioxidants alone. These ferroptotic cascades are intimately linked to other RCD pathways—including pyroptosis, autophagy, and apoptosis—creating a complex, self-amplifying network of neurodegeneration (Tian et al., 2024; Li et al., 2025).

The convergence of these pathogenic mechanisms has spurred intensive efforts to develop therapeutic strategies targeting ferroptosis in PD. Multiple classes of ferroptosis inhibitors, including radical-trapping antioxidants (ferrostatin-1, liproxstatin-1), iron chelators (deferoxamine, deferiprone), and modulators of the Nrf2-GPX4 axis, have demonstrated neuroprotective efficacy in rotenone models (Hirata et al., 2022; Hu et al., 2023). Moreover, several FDA-approved drugs with established safety profiles—such as carvedilol, lapatinib, and pazopanib—have been repurposed as ferroptosis inhibitors, offering accelerated pathways to clinical translation (Mansour et al., 2023a; Mansour et al., 2023b; Abuelezz et al., 2025). However, approved status in other indications does not ensure sufficient brain exposure, disease-relevant dosing, or nigrostriatal target engagement. Carvedilol is brain-penetrant but may be limited by β-blocking and hemodynamic effects in elderly PD patients; lapatinib and pazopanib are oncology agents with dose-limiting systemic toxicities and uncertain CNS exposure at ferroptosis-inhibiting doses; and idebenone has variable bioavailability and brain penetration (Avcı et al., 2020; Mansour et al., 2023a; Mansour et al., 2023b; Abuelezz et al., 2025; Cheng et al., 2026). Therefore, accelerated translation remains hypothetical until CNS exposure, pharmacodynamic biomarkers, and chronic safety are established in PD-relevant models and early-phase trials.

In this review, we systematically examine the molecular mechanisms by which rotenone induces ferroptosis in dopaminergic neurons, the crosstalk between ferroptosis and other RCD pathways, and the therapeutic implications of targeting ferroptosis in PD. We further discuss current challenges, future directions, and the translational potential of ferroptosis-based strategies in the treatment of this devastating disorder.

## 2 Ferroptosis

Ferroptosis is a regulated form of oxidative, iron-dependent cell death, driven primarily by the peroxidation of PUFA tails within membrane phospholipids (Figure 1) (Dixon et al., 2012). It was initially characterized by its activation via specific small molecules, including erastin—an irreversible inhibitor of the cysteine/glutamate antiporter SLC7A11 (or xCT)—and RSL3, which directly inhibits the phospholipid hydroperoxidase GPX4 (Yang and Stockwell, 2008). Morphologically and mechanistically distinct from apoptosis and necroptosis, ferroptosis does not engage the core protein machinery that governs these canonical programmed pathways (Yang and Stockwell, 2008). Whereas apoptosis, necroptosis, and pyroptosis are triggered by specific physiological cues and rely on dedicated protein complexes, ferroptosis arises from the breakdown of cellular antioxidant defenses and is subject to metabolic regulation (Dixon et al., 2012). This process is intimately linked to amino acid and iron metabolism—including absorption, storage, utilization, and efflux—as well as phospholipid biosynthesis (Dixon et al., 2012; Stockwell, 2022).

**FIGURE 1:**
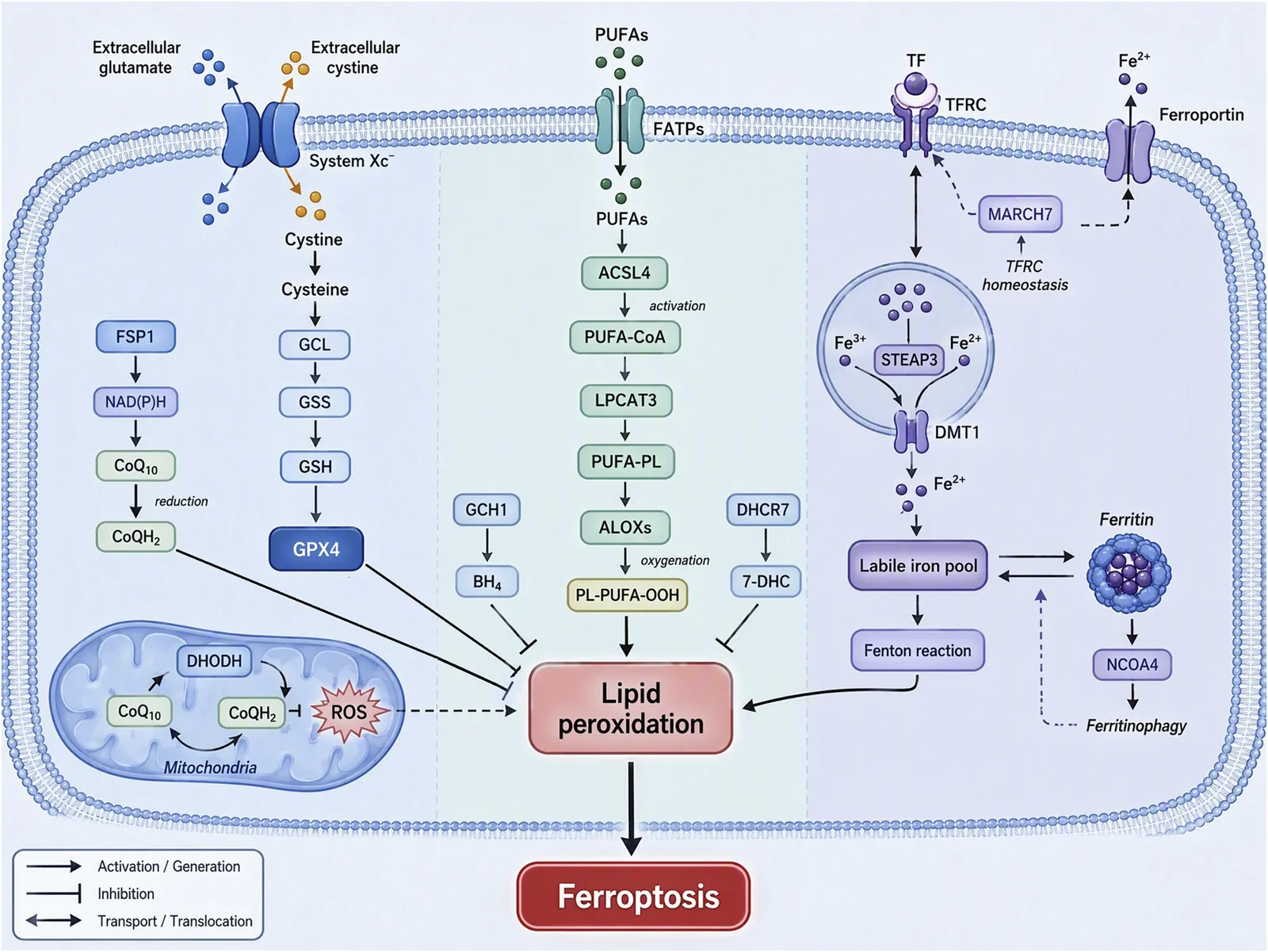
Molecular mechanisms of ferroptosis. Ferroptosis is an iron-dependent, oxidative form of regulated cell death driven by lethal peroxidation of polyunsaturated fatty acid (PUFA)-containing phospholipids. The primary antioxidant defense is the System Xc^−^–GPX4 axis: System Xc^−^ (SLC7A11/SLC3A2) imports cystine for glutathione (GSH) synthesis, and GPX4 uses GSH to reduce phospholipid hydroperoxides. Parallel surveillance systems include the CoQ_10_–FSP1 and DHODH pathways, which regenerate ubiquinol, a lipophilic radical-trapping antioxidant, and the GCH1–BH4 pathway. Lipid peroxidation is catalyzed by enzymes such as lipoxygenases (LOXs), POR, and CYB5R1, and is amplified by ferrous iron (Fe^2+^) via the Fenton reaction. ACSL4 and LPCAT3 promote PUFA incorporation into membranes, favoring ferroptosis, whereas SCD1, ACSL3, and MBOAT1/2 promote monounsaturated fatty acid (MUFA) incorporation and resistance. Mitochondria contribute to ROS production under certain conditions. The ESCRT-III membrane repair system can delay ferroptosis by removing peroxidized membrane patches. Polyamines (e.g., spermine) act as endogenous iron chelators, and non-canonical pathways such as the GPX1–OSBPL8 axis also suppress ferroptosis *in vivo*.

Cellular susceptibility to ferroptosis is largely dictated by the abundance of PUFA-containing lipids, which serve as critical substrates for peroxidation (Kagan et al., 2017). This peroxidation generates reactive aldehydes, notably 4-hydroxynonenal (4-HNE), which form adducts with proteins, lipids, and DNA, ultimately compromising plasma and mitochondrial membrane integrity and culminating in cell death (Gentile et al., 2017; Vazdar et al., 2021; Zhang et al., 2021). Key enzymes involved in the biosynthesis of PUFA-containing phospholipids, such as ACSL4 and LPCAT3, are essential for ferroptosis execution (Doll et al., 2017). In contrast, enzymes that promote the synthesis of monounsaturated fatty acid (MUFA)-containing lipids—including SCD1, ACSL3, and MBOAT1/2—antagonize PUFA incorporation, thereby attenuating ferroptotic sensitivity (Magtanong et al., 2019; Liang et al., 2023).

The primary defense against lipid peroxidation is orchestrated by the System Xc^−^–GPX4 axis (Dixon et al., 2012; Yang et al., 2014). System Xc^−^, a heterodimeric transporter composed of SLC7A11 and SLC3A2, mediates the uptake of cystine (the oxidized form of cysteine in exchange for intracellular glutamate. Once imported, cystine is reduced to cysteine, which serves as a rate-limiting precursor for glutathione (GSH) biosynthesis. GPX4) is the sole known phospholipid hydroperoxidase that utilizes GSH to reduce phospholipid hydroperoxides, thereby safeguarding cellular membranes from oxidative injury (Dixon et al., 2012; Yang et al., 2014). Beyond this core axis, parallel surveillance mechanisms have been identified, including the CoQ10–FSP1 and CoQ10–DHODH axes (Bersuker et al., 2019; Doll et al., 2019; Mao et al., 2021). Both FSP1 and DHODH catalyze the reduction of oxidized coenzyme Q10 (ubiquinone) to its reduced form, ubiquinol, a lipophilic radical-trapping antioxidant. In addition, FSP1 reduces vitamin K to its hydroquinone form, which similarly exerts antioxidant activity (Mishima et al., 2022). Furthermore, tetrahydrobiopterin (BH4) biosynthesis, involving GCH1 and other enzymes, generates another radical-trapping antioxidant that contributes to protection against lipid peroxidation (Kraft et al., 2020; Soula et al., 2020). These systems act in a complementary manner, with individual components assuming dominant roles in specific tissues or cell types. In addition to these canonical pathways, recent studies have uncovered novel layers of ferroptosis regulation. March7 has been identified as a “ferro-guardian” that stabilizes GPX4 protein levels, and structural analyses have revealed that a fin-loop-like motif within GPX4 is essential for its neuroprotective function (Huang et al., 2026; Lorenz et al., 2026). This E3 ligase-mediated stabilization adds an additional regulatory tier to the GPX4-dependent surveillance system, particularly relevant under oxidative stress conditions where GPX4 is prone to degradation (Huang et al., 2026). Moreover, non-canonical surveillance systems—such as the GPX1–OSBPL8 axis—operate independently of GPX4 to suppress ferroptosis *in vivo* (Xia et al., 2026). This pathway has been shown to inhibit lipid peroxidation and tumor growth, thereby expanding the anti-ferroptotic network beyond classical models and highlighting the regulatory complexity of ferroptosis in both physiological and pathological contexts (Xia et al., 2026). At the level of iron metabolism, polyamines—particularly spermine—function as endogenous iron chelators that buffer labile ferrous iron, thus limiting Fenton chemistry and ferroptosis execution (Li et al., 2026; Sharma et al., 2026). Collectively, these discoveries broaden the conceptual framework of ferroptosis defense from a single antioxidant axis into a multi-layered, integrated network encompassing protein stability, structural biology, metabolic pathways, and non-canonical effectors.

Lipid peroxidation is driven by reactive oxygen species (ROS), which are generated primarily through electron leakage from several enzymatic sources, including lipoxygenases (LOXs), the mitochondrial electron transport chain, NADPH oxidase, and the endoplasmic reticulum (ER)-resident enzymes NADPH–cytochrome P450 reductase (POR) and CYB5R1 (Wenzel et al., 2017; Chu et al., 2019; Gao et al., 2019; Zou et al., 2020; Yan et al., 2021). These enzymes reduce oxygen to hydrogen peroxide, which is subsequently oxidized by ferrous iron (Fe^2+^) via the Fenton reaction to produce highly reactive hydroxyl radicals that initiate peroxidative chain reactions in membrane lipids (Yan et al., 2021). Beyond protein-based iron sequestration (ferritin) and export (ferroportin), cells employ small-molecule metabolites to fine-tune iron bioavailability. In this context, polyamines—particularly spermidine and spermine—act as endogenous chelators that buffer labile ferrous iron pools, thereby constraining Fenton chemistry and suppressing ferroptosis (Sharma et al., 2026). Spermine, in particular, has been characterized as a potent natural iron-chelating metabolite that effectively inhibits ferroptotic cell death, highlighting its potential relevance in neurodegenerative disorders marked by iron dyshomeostasis, such as Parkinson’s disease (Li et al., 2026). Given that the ER serves as the primary site for PUFA incorporation and houses both POR and CYB5R1, it likely represents the initial locus of lipid peroxidation, with plasma membrane damage occurring as a later event (Yan et al., 2021). Mitochondria also contribute to lipid peroxide production during cysteine-deprivation-induced ferroptosis, although they appear dispensable when GPX4 is directly inhibited (Gao et al., 2019). Finally, while the attainment of a critical threshold—approximately 2% peroxidized membrane lipids—appears to precipitate membrane rupture, the precise molecular mechanisms by which lipid peroxidation culminates in physical membrane disintegration remain incompletely understood (von Krusenstiern et al., 2023).

Unlike apoptosis, necroptosis, and pyroptosis—each of which serves well-defined roles in development and immunity—ferroptosis results from the breakdown of homeostatic redox regulation. It has been implicated in degenerative diseases affecting multiple organ systems and is actively suppressed during tumor initiation and progression (Gao et al., 2022). Notably, ferroptosis operates independently of the core apoptotic or necroptotic machinery (i.e., caspases, RIPKs, or MLKL) and exhibits distinct morphological features. Ferroptosis also engages in crosstalk with other cell death pathways. For instance, interferon-γ (IFNγ) promotes ferroptosis by downregulating SLC7A11 and SLC3A2, thereby curtailing cystine uptake and glutathione synthesis (Wang et al., 2019). Conversely, the ESCRT-III membrane repair system can delay ferroptosis by removing peroxidized lipid-damaged membrane patches (Dai et al., 2020). A comprehensive understanding of ferroptosis—through its regulation of lipid metabolism, iron homeostasis, and antioxidant defenses—provides a rational basis for therapeutic targeting in diseases spanning neurodegeneration to cancer (Wang et al., 2023a; Wang et al., 2023b; Wang et al., 2023c; Song et al., 2026).

## 3 Rotenone

### 3.1 Basic knowledge of rotenone

Rotenone ([2R-(2α,6aα,12aα)]-1,2,12,12a-tetrahydro-8,9-dimethoxy-2-(1-methylethenyl)-[1] benzopyran [3,4-b]furo [2,3-h][1]benzopyran-6 (6aH)-one) is a naturally occurring compound derived from the roots, seeds, and stems of tropical plants belonging to the Leguminosae family, including *Derris*, *Tephrosia*, *Lonchocarpus*, and *Mundulea* species (Radad et al., 2019). It belongs to a class of compounds known as isoflavones (Radad et al., 2019). Rotenone is an odorless, colorless to brownish crystalline powder with an empirical formula of C_23_H_22_O_6_ and a molecular weight of 394.41 g/mol. It is insoluble in water but highly soluble in many organic solvents (Radad et al., 2019). Rotenone is highly sensitive to light and oxygen, decomposing to less toxic products such as rotenolone; the rate of decomposition depends on temperature, pH, sunlight, depth, dose, and the presence of organic debris. Rotenone has been used for centuries—Peruvian natives used crude extracts of rotenone-containing plants to catch fish. It was first registered as a pesticide in the USA in 1947 and was widely used in agriculture and households until its registration was withdrawn in 2006. It acts as a broad-spectrum insecticide, pesticide, and piscicide (Amaral et al., 2025). Rotenone is absorbed through the gastrointestinal tract, lungs, and skin, and because it is lipophilic, it readily penetrates the brain.

Primary mechanism of action: Rotenone is a potent inhibitor of mitochondrial electron transport at NADH:ubiquinone oxidoreductase (mitochondrial complex I) (Ibarra-Gutiérrez et al., 2023). It disrupts electron transfer between NADH and coenzyme Q (ubiquinone) in the inner mitochondrial membrane.

### 3.2 Research history of rotenone in PD

The insecticidal properties of rotenone were first reported in the 1840s, when it was observed that caterpillars eating rotenone-producing plant leaves showed immediate effects. Rotenone was first isolated between 1895 and 1902—French botanist Emmanuel Geoffroy isolated a compound he called “nicouline” from *Lonchocarpus nicou*, while Japanese chemist Nagai Nagayoshi isolated a pure crystalline compound from *Derris elliptica* and named it “rotenone” in 1902. The two compounds were recognized as chemically identical by 1930.

In the early 1980s, the first toxin found to induce parkinsonism was 1-methyl-4-phenyl-1,2,3,6-tetrahydropyridine (MPTP), a contaminant of synthetic heroin that caused severe parkinsonism in humans (Mogi et al., 1987). MPTP’s active metabolite, MPP^+^, was found to inhibit mitochondrial complex I. This discovery led researchers to investigate whether other complex I inhibitors could produce similar effects (Wen et al., 2020; Thirugnanam and Santhakumar, 2022). In Heikkila et al. (1985) first used rotenone in experimental paradigms related to parkinsonism (Casanova et al., 2022). However, it was not until the late 1990s that laboratories began developing the rotenone model more systematically, driven by growing suspicion that Parkinson’s disease might be associated with systemic mitochondrial defects. The pivotal moment came in 2000 when Betarbet et al. (2000) published a landmark study in *Nature Neuroscience* demonstrating that chronic, systemic administration of rotenone in rats reproduced most of the behavioral, biochemical, and pathological features of PD. The rotenone-treated rats exhibited selective nigrostriatal dopaminergic degeneration, formation of cytoplasmic inclusions containing α-synuclein and ubiquitin (resembling Lewy bodies), and behavioral features including hypokinesia and rigidity (Betarbet et al., 2000). This study established rotenone as a valuable experimental model for investigating the mechanisms underlying PD and evaluating potential therapies (Thirugnanam and Santhakumar, 2022; Lal et al., 2024; Amaral et al., 2025).

Epidemiological evidence has supported the link between rotenone exposure and PD risk. A study in eastern Texas found that household gardening use of rotenone increased the risk of PD with an odds ratio of 10.9 (95% CI 2.9–34.3) (Kanthasamy et al., 2019). The AGRICAN cohort study also reported an increased risk of PD associated with rotenone use (OR = 1.57), especially in farmers with the longest exposure. However, some researchers have noted that available epidemiological data do not provide fully convincing evidence for a causal association between rotenone exposure and PD in humans.

## 4 Potential role of rotenone-induced ferroptosis in PD pathogenesis: preclinical evidence and caveats

This section aims to systematically dissect the multifaceted molecular circuitry through which rotenone precipitates ferroptosis in dopaminergic neurons, thereby driving Parkinsonian neurodegeneration (Figure 2). Throughout this section, we distinguish established mechanisms, defined as those supported by direct pharmacological or genetic evidence in rotenone models, from proposed mechanisms, defined as those inferred from correlative data, context-dependent findings, or evidence primarily from other PD models. Rather than operating through a single linear pathway, rotenone engages a coordinated network of pathological cascades that converge on lipid peroxidation and iron-dependent death. We will first examine how rotenone disrupts iron homeostasis, leading to ferrous iron accumulation and Fenton-driven oxidative damage, and then detail the inactivation of the System Xc^−^–GPX4 antioxidant axis—the primary defense against ferroptosis. Subsequent subsections address rotenone-induced dysregulation of lipid metabolism that enriches pro-ferroptotic membrane substrates, mitochondrial dysfunction that amplifies ROS production, ferritinophagy-mediated iron release that propagates ferroptotic signals, and the interplay with neuroinflammation and SIRT1–Nrf2 pathway suppression. Finally, we explore the crosstalk between ferroptosis and apoptosis, underscoring that these death modalities do not operate in isolation. By integrating these mechanisms, we aim to establish a comprehensive framework for understanding how rotenone triggers ferroptosis and to identify potential nodes for therapeutic intervention.

**FIGURE 2:**
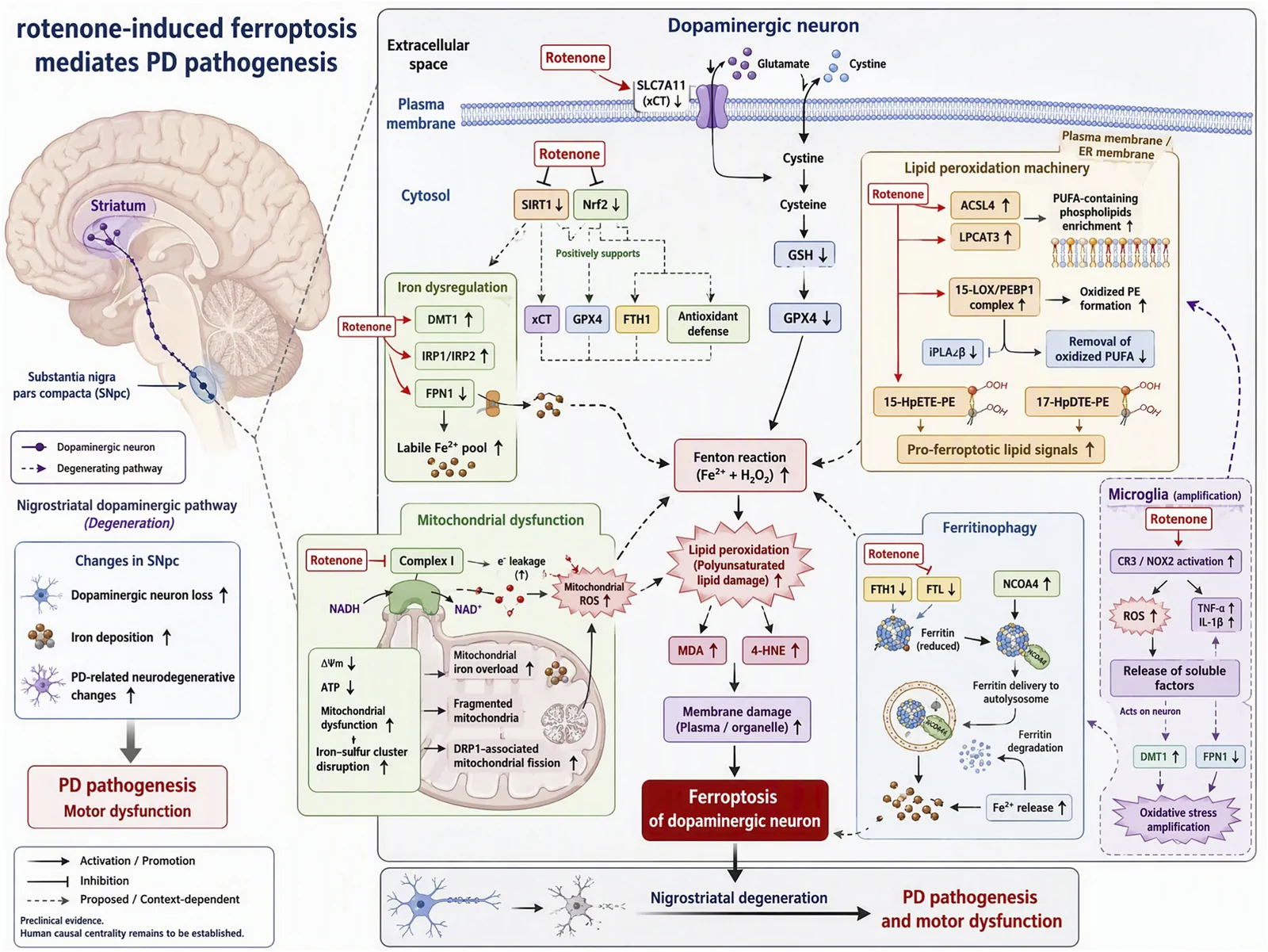
Multifaceted ferroptotic cascades induced by rotenone in dopaminergic neurons. Rotenone, a mitochondrial complex I inhibitor, triggers ferroptosis through interconnected pathological pathways that converge on lipid peroxidation and iron-dependent cell death. 2-2-6 Solid arrows indicate established or strongly supported links in rotenone models; dashed arrows indicate proposed, context-dependent, or not-yet-validated links. Mitochondrial iron-sulfur cluster release, DRP1-dependent fission, CR3/NOX2-mediated glial–neuronal crosstalk, and apoptosis–ferroptosis crosstalk are presented as proposed amplifiers rather than fully established obligatory mechanisms. (1) Iron dyshomeostasis: Rotenone upregulates DMT1 (iron import) and downregulates FPN1 (iron export), increasing labile Fe^2+^ pools; mitochondrial iron-sulfur cluster destabilization releases additional free iron, fueling Fenton chemistry. (2) Suppression of the System Xc^−^–GPX4 axis: Rotenone reduces SLC7A11 expression, depleting GSH, and decreases GPX4 via transcriptional (SIRT1–Nrf2 inhibition) and post-translational degradation, allowing lipid hydroperoxide accumulation. (3) Lipid metabolism dysregulation: Upregulation of ACSL4 and LPCAT3 enriches membrane phospholipids with oxidation-prone PUFAs; 15-LOX/PEBP1 generates pro-ferroptotic signals (15-HpETE-PE and 17-HpDTE-PE), while iPLA2β dysfunction prevents their clearance. (4) Mitochondrial dysfunction: ROS overproduction, loss of ΔΨm, and impaired mitophagy amplify oxidative damage; DRP1-mediated fission promotes ferroptosis. (5) Ferritinophagy: NCOA4 mediates autophagic degradation of ferritin, releasing iron that perpetuates lipid peroxidation. (6) Neuroinflammation: Microglial CR3/NOX2 activation produces ROS and cytokines (TNFα, IL-1β) that further disrupt neuronal iron homeostasis and ferroptosis. (7) SIRT1–Nrf2 suppression: Rotenone inhibits this protective axis, reducing expression of GPX4, SLC7A11, FTH1, and HO-1. (8) Apoptosis–ferroptosis crosstalk: ROS and BAX-mediated mitochondrial permeabilization link both death pathways. This integrated network drives progressive dopaminergic neurodegeneration.

### 4.1 Disruption of iron homeostasis and iron-dependent lipid peroxidation

Iron dyshomeostasis constitutes the fundamental trigger of ferroptosis in rotenone-induced PD models. Rotenone exposure induces significant iron accumulation in the substantia nigra and striatum, with iron deposition occurring as an early event preceding overt dopaminergic neuron loss (Ma et al., 2024). This spatial and temporal pattern exhibits remarkable regional selectivity: the substantia nigra and striatum show iron deposition as early as 2 weeks after intragastric rotenone administration, whereas regions such as the ventral tegmental area are relatively resistant (Ma et al., 2024). These data establish early iron dyshomeostasis and iron-dependent lipid peroxidation, but not ferroptosis *per se*; ferroptosis specificity is supported by GPX4 inactivation, ACSL4/LPCAT3-dependent PUFA-PE peroxidation, NCOA4-mediated ferritinophagy, and reversal by Fer-1/DFO. The selective vulnerability of nigrostriatal dopaminergic neurons reflects their intrinsically high iron demand for dopamine synthesis and the presence of redox-active dopamine metabolites that amplify oxidative damage.

At the molecular level, rotenone-induced iron accumulation involves coordinated alterations in iron transport and regulatory proteins. Rotenone significantly upregulates divalent metal transporter 1 (DMT1), the principal iron importer, while downregulating ferroportin 1 (FPN1), the sole cellular iron exporter (Ma et al., 2024; Sun et al., 2024). This reciprocal dysregulation favors cellular iron retention. Importantly, these changes are not necessarily independent events. Iron regulatory proteins IRP1 and IRP2, which post-transcriptionally control iron metabolism genes, are elevated after rotenone exposure (Sun et al., 2024; Liu et al., 2025). Through the IRP/IRE system, IRP activation can stabilize transcripts such as transferrin receptor 1 and DMT1 while inhibiting translation of FPN1 and ferritin. Thus, DMT1 upregulation, FPN1 downregulation, and ferritin suppression can be viewed as coordinated outputs of a shared upstream regulatory response, although additional rotenone-sensitive pathways may also contribute. The functional consequence is a marked increase in labile ferrous iron (Fe^2+^) pools within dopaminergic neurons, as demonstrated by elevated FerroOrange and RhoNox-1 staining in rotenone-treated cells and tissues (Sun et al., 2024; Liu et al., 2025). These probes provide useful evidence of labile Fe^2+^ accumulation, but they should be interpreted with the caveat that they do not fully resolve subcellular iron distribution or distinguish cytosolic from mitochondrial pools. More compartment-resolved approaches, such as mitochondria-targeted Fe^2+^ probes, ICP-MS, or synchrotron-based elemental imaging, would strengthen future mechanistic conclusions Ferritin dynamics provide an important link between iron storage, autophagy, and ferroptosis. Ferritin heavy chain 1 (FTH1) possesses ferroxidase activity and is critical for safe iron sequestration, whereas ferritin light chain (FTL) contributes to iron mineralization and ferritin stability. In rotenone-induced neurotoxicity, NCOA4 functions as the cargo receptor that delivers ferritin to autophagosomes for lysosomal degradation, releasing stored iron (Sun et al., 2024). Rotenone exposure upregulates NCOA4 and increases LC3-II/LC3-I ratios and ATG5 expression, indicating enhanced autophagic flux that facilitates ferritin delivery to lysosomes (Sun et al., 2024; Li et al., 2025). The downstream consequence is reduced FTH1 expression, loss of ferritin’s iron-sequestering capacity, and subsequent free iron accumulation (Sun et al., 2024). Double immunofluorescence confirms co-localization of NCOA4 and tyrosine hydroxylase in dopaminergic neurons, establishing cell-type specificity (Sun et al., 2024). This ferritinophagy pathway is therefore best understood as a sequential autophagic mechanism that occurs downstream of autophagy activation but upstream of the labile iron surge. However, the pro-ferroptotic role of ferritinophagy must be interpreted within the broader context of autophagy, which can be either protective or deleterious depending on cargo selectivity and cellular state. General autophagy promotes survival by clearing damaged mitochondria, protein aggregates, and oxidized macromolecules, whereas NCOA4-mediated ferritinophagy selectively delivers ferritin to autophagosomes for lysosomal degradation, thereby liberating stored iron and amplifying labile Fe^2+^ and lipid peroxidation (Sun et al., 2024; Abuelezz et al., 2025). In rotenone models, chloroquine, which blocks autophagosome–lysosome fusion, attenuates ferritin degradation and ferroptosis, suggesting that autolysosomal ferritin breakdown is a major iron source (Sun et al., 2024). However, because chloroquine inhibits general autophagy, its net benefit may be context-dependent and could be offset by impaired clearance of damaged mitochondria and α-synuclein, particularly with prolonged treatment (Sun et al., 2024; Abuelezz et al., 2025). Conversely, rapamycin-induced autophagy activation exacerbates rotenone-induced ferroptosis, consistent with enhanced ferritinophagy and iron release (Sun et al., 2024). Yet rapamycin can also promote mitophagy and mitochondrial quality control in other settings, so its effect on neuronal survival depends on the relative balance between selective ferritinophagy and protective mitophagy (Sun et al., 2024; Abuelezz et al., 2025). Increased LC3-II/LC3-I ratios and ATG5 expression indicate enhanced autophagic flux but do not establish selective ferritin degradation, because these are general autophagy markers/effectors (Sun et al., 2024; Abuelezz et al., 2025). More specific evidence comes from NCOA4 manipulation: NCOA4 knockdown reduces rotenone-induced iron accumulation and lipid peroxidation, whereas NCOA4 overexpression counteracts ferroptosis inhibitors (Sun et al., 2024). Clozapine-N-oxide protects dopaminergic neurons by inhibiting NCOA4-mediated ferritinophagy without globally suppressing autophagy or mitophagy, supporting the idea that selective ferritinophagy, rather than autophagy *per se*, is the pro-ferroptotic node (Sun et al., 2024). Additional work shows that H_2_S/ISM1 signaling inhibits ferritinophagy-mediated ferroptosis and preserves GPX4/xCT, further separating selective ferritinophagy from general autophagic flux (Linjacki et al., 2024; Chen et al., 2025). Thus, future studies should combine NCOA4–FTH1 co-localization, ferritin flux, labile iron measurements, and mitophagy readouts to determine whether autophagy is protective or detrimental in a given rotenone context (Ma et al., 2024; Sun et al., 2024; Abuelezz et al., 2025; Liu et al., 2025). The differential degradation of FTH1 versus FTL and the contribution of mitochondrial iron overload also remain unresolved and should be addressed with compartment-resolved approaches (Ma et al., 2024; Sun et al., 2024; Liu et al., 2025). At the same time, IRP-mediated suppression of ferritin translation and NCOA4-mediated ferritin degradation act at different levels, translational and autophagic, to converge on reduced iron storage. A more critical unresolved issue is the differential dynamics of FTH1 and FTL. Most current evidence focuses on FTH1, while FTL behavior, the FTH1/FTL ratio, and whether ferritin subunits are degraded synchronously remain incompletely defined.

Mitochondrial iron overload represents a particularly deleterious and partially parallel event. Rotenone-induced mitochondrial dysfunction, characterized by impaired electron transport chain activity and reduced membrane potential, disrupts mitochondrial iron-sulfur cluster biogenesis (Lin et al., 2026). Iron-sulfur clusters are essential cofactors for mitochondrial respiratory complexes, and their destabilization releases free iron into the mitochondrial matrix, establishing a vicious cycle of mitochondrial iron accumulation and oxidative damage (Linjacki et al., 2024). Transmission electron microscopy reveals mitochondrial shrinkage, increased membrane density, cristae disruption, and outer membrane rupture, which are ultrastructural hallmarks of ferroptotic cell death (Hu et al., 2023; Sun et al., 2024). Mitochondrial iron handling involves proteins such as mitoferrin, ABCB7, frataxin, and mitochondrial ferritin, but how these are altered in rotenone models requires further clarification. Thus, mitochondrial iron overload can be viewed as both a consequence of early mitochondrial dysfunction and an amplification loop that feeds back into cytosolic labile iron and lipid peroxidation.

Considered together, these mechanisms are best conceptualized as a temporally ordered but feed-forward network rather than as strictly parallel or strictly sequential events. Early rotenone exposure promotes IRP activation, DMT1 upregulation, FPN1 downregulation, and ferritin suppression, leading to increased cytosolic labile iron. In parallel, mitochondrial dysfunction impairs iron-sulfur cluster biogenesis and promotes mitochondrial iron retention. Subsequently, NCOA4-mediated ferritinophagy releases additional iron from ferritin stores, further expanding the labile iron pool. These pathways converge on ferrous iron, which participates in the Fenton reaction with hydrogen peroxide to generate hydroxyl radicals. These radicals initiate peroxidation of polyunsaturated fatty acid-containing phospholipids, particularly arachidonic acid- and adrenic acid-containing phosphatidylethanolamines (Sun et al., 2021). Lipidomics analyses have identified 15-hydroperoxy-arachidonoyl-phosphatidylethanolamine and 17-hydroperoxy-docosatetraenoyl-phosphatidylethanolamine as specific pro-ferroptotic death signals that accumulate in rotenone-exposed neurons (Sun et al., 2021; Wang et al., 2024). These oxidized phospholipids compromise membrane integrity and execute ferroptotic cell death.

The functional significance of ferritinophagy is supported by pharmacological and genetic interventions. Chloroquine, a lysosomal inhibitor that blocks autophagosome-lysosome fusion, attenuates rotenone-induced ferritin degradation and ferroptosis, indicating that the autolysosomal pathway mediates ferritin degradation (Sun et al., 2024). Conversely, rapamycin-induced autophagy activation exacerbates rotenone-induced ferroptosis, consistent with ferritinophagy promoting iron release (Sun et al., 2024). NCOA4 knockdown using siRNA attenuates rotenone-induced iron accumulation and lipid peroxidation, whereas NCOA4 overexpression counteracts the protective effects of ferroptosis inhibitors (Sun et al., 2024). These findings establish ferritinophagy as an upstream regulator of iron-dependent ferroptosis in rotenone-induced PD models. Overall, the revised integrated framework clarifies that DMT1, FPN1, IRP1/IRP2, ferritin, mitochondrial iron overload, and NCOA4-mediated ferritinophagy are interconnected through early transport dysregulation, autophagic iron release, and mitochondrial amplification, ultimately converging on labile Fe^2+^-driven lipid peroxidation and ferroptotic dopaminergic neuron death.

### 4.2 Inactivation of the system Xc^−^-GPX4 antioxidant axis

The System Xc^−^–GPX4 axis is a primary defense against ferroptosis, but rotenone perturbs this axis at multiple levels rather than through a single linear GPX4-suppression cascade. Rotenone consistently reduces xCT/SLC7A11 and depletes GSH (Sun et al., 2024; Li et al., 2025), which can impair GPX4 activity by limiting its GSH substrate even before GPX4 protein levels change. GPX4 downregulation in rotenone models has been attributed to both transcriptional and post-translational mechanisms (Sun et al., 2024; Liu et al., 2025). Transcriptional repression may involve inhibition of SIRT1–Nrf2 signaling, because Nrf2 can transactivate GPX4 through antioxidant-response elements (Liu et al., 2025; Ma et al., 2026); however, direct chromatin-level evidence in rotenone-treated dopaminergic neurons remains limited, and GPX4 mRNA and protein changes are not always concordant (Sun et al., 2024; Liu et al., 2025). Post-translationally, GPX4 is sensitive to oxidative stress and can be degraded via proteasomal and lysosomal routes, although the relative contribution of each route in rotenone models is not firmly established (Sun et al., 2024; Liu et al., 2025). GPX4 protein stability is also actively maintained by March7, an E3 ligase described as a ferro-guardian that stabilizes GPX4 (Huang et al., 2026), and structural features such as the fin-loop-like motif are required for its neuroprotective function (Lorenz et al., 2026). In addition, non-canonical GPX1–OSBPL8 signaling can suppress ferroptosis independently of GPX4 *in vivo* (Xia et al., 2026), indicating that GPX4 loss is not the sole determinant of ferroptotic sensitivity. Accordingly, rotenone-induced GPX4 suppression should be interpreted as a context-dependent outcome of impaired transcription, accelerated degradation, and reduced substrate availability, with compensatory or parallel antioxidant systems potentially modifying the phenotype (Sun et al., 2024; Liu et al., 2025; Huang et al., 2026; Lorenz et al., 2026; Ma et al., 2026; Xia et al., 2026). Pharmacological restoration of GPX4 by Fer-1 or DFO (Sun et al., 2024; Liu et al., 2025) and phenocopy by RSL3 (Sun et al., 2024) support the importance of this axis, but do not establish a single obligatory linear pathway.

### 4.3 Lipid metabolism dysregulation and accumulation of pro-ferroptotic signals

The esterification of PUFAs into membrane phospholipids is a prerequisite for ferroptotic lipid peroxidation, and rotenone activates the enzymatic machinery responsible for generating pro-ferroptotic lipid substrates. ACSL4, which catalyzes the CoA-dependent esterification of arachidonic and adrenic acids, is significantly upregulated in rotenone-treated neurons (Hu et al., 2023; Mansour et al., 2023a; Sun et al., 2024). ACSL4 expression is elevated through both transcriptional activation and post-translational mechanisms involving the PKCβII signaling pathway (Mansour et al., 2023a). The functional significance of ACSL4 upregulation is demonstrated by the observation that ACSL4 knockdown or inhibition attenuates rotenone-induced ferroptosis, highlighting ACSL4 as a critical determinant of ferroptosis sensitivity (Mansour et al., 2023a).

LPCAT3 mediates the re-acylation of lysophosphatidylethanolamine with arachidonoyl-CoA, generating the membrane phospholipid substrates for lipoxygenase-mediated oxidation. Rotenone exposure upregulates LPCAT3 expression, promoting the incorporation of arachidonic acid into phosphatidylethanolamine species (Sun et al., 2024). The coordinated activation of ACSL4 and LPCAT3 enriches membrane phospholipids with oxidation-prone PUFA substrates, creating a permissive environment for ferroptotic lipid peroxidation (Sun et al., 2021).

The enzymatic oxidation of PUFA-containing phospholipids is catalyzed by 15-lipoxygenase (15-LOX) complexed with phosphatidylethanolamine-binding protein 1 (PEBP1). This enzymatic complex specifically generates 15-HpETE-PE and 17-HpDTE-PE, the proximate executioners of ferroptotic cell death (Sun et al., 2021). In rotenone-treated neurons, elevated 15-LOX activity and increased PEBP1 expression promote the accumulation of these pro-ferroptotic lipid signals (Sun et al., 2021). Calcium-independent phospholipase A2β (iPLA2β) normally serves as a protective mechanism by hydrolyzing oxidized phospholipids, thereby eliminating pro-ferroptotic signals (Sun et al., 2021). However, rotenone-induced iPLA2β dysfunction, either through reduced expression or impaired catalytic activity, compromises this protective pathway, allowing 15-HpETE-PE accumulation and ferroptosis propagation (Sun et al., 2021).

The lipid peroxidation cascade culminates in the formation of reactive aldehydes, including MDA and 4-HNE, which form adducts with proteins and DNA, propagating cellular damage (Hu et al., 2023; Mansour et al., 2023a; Ma et al., 2024). Elevated MDA and 4-HNE levels are consistently observed in rotenone-treated cells and brain tissues, serving as reliable biomarkers of ferroptotic lipid peroxidation (Hu et al., 2023; Mansour et al., 2023a). The correlation between lipid peroxidation markers and motor deficits in rotenone-treated mice underscores the pathophysiological significance of this pathway (Hu et al., 2023; Tian et al., 2024).

The pro-ferroptotic and protective lipid pools are chemically and enzymatically distinct. PUFA-containing phosphatidylethanolamines (PE), especially arachidonic acid (C20:4)- and adrenic acid (C22:4)-PE, are the preferred substrates for peroxidation and are generated by ACSL4 and LPCAT3(Doll et al., 2017; Kagan et al., 2017; Sun et al., 2024). In rotenone-treated neurons, ACSL4 and LPCAT3 upregulation enriches these oxidation-prone PE species, while 15-LOX/PEBP1 converts them into 15-HpETE-PE and 17-HpDTE-PE, the proximate death signals (Sun et al., 2021; Mansour et al., 2023a; Sun et al., 2024). In contrast, MUFA-containing lipids, such as oleic acid (C18:1)-containing phospholipids, are poor peroxidation substrates and compete with PUFAs for incorporation into membrane phospholipids. SCD1, ACSL3, and MBOAT1/2 promote MUFA synthesis and esterification, thereby reducing PUFA-PE abundance and conferring ferroptosis resistance (Magtanong et al., 2019; Liang et al., 2023). Thus, the ACSL4/LPCAT3 versus SCD1/ACSL3/MBOAT1/2 balance, rather than total lipid content, determines the ferroptotic threshold. Rotenone appears to shift this balance toward PUFA-rich, MUFA-poor membranes, although direct measurements of PUFA/MUFA ratios in the substantia nigra are still limited (Hu et al., 2023; Mansour et al., 2023a; Sun et al., 2024).

These lipidomic alterations have translational potential as biomarkers. Oxidized phospholipid species such as 15-HpETE-PE and 17-HpDTE-PE are chemically stable enough to be detected by targeted LC-MS/MS in biofluids or postmortem tissue and could serve as proximal ferroptosis markers, whereas MDA and 4-HNE are widely used but less specific because they also reflect general oxidative stress (Sun et al., 2021; Hu et al., 2023; Mansour et al., 2023; Ma et al., 2024). A PUFA/MUFA lipidomic signature, combined with GPX4 activity, ferritin, and labile iron measures, may improve patient stratification and pharmacodynamic monitoring for ferroptosis-targeted therapies (Stockwell, 2022; von Krusenstiern et al., 2023). However, current evidence is largely preclinical, and standardized preanalytical protocols, reference ranges, and validation in idiopathic PD cohorts are needed before clinical implementation (Stockwell, 2022; Liu et al., 2025).

Cell-type-specific lipid profiles in the substantia nigra are likely important. Dopaminergic neurons have high PUFA demand for membrane remodeling and are intrinsically vulnerable to iron-dependent lipid peroxidation, whereas astrocytes and microglia may have different PUFA/MUFA compositions and antioxidant capacities (Ma et al., 2024; Tian et al., 2024; Cheng et al., 2026). Rotenone-induced iron deposition and ferroptosis are regionally selective for the substantia nigra and striatum, and microglial CR3/NOX2 activation further shapes the neuronal lipid environment through cytokines and ROS (Ma et al., 2024; Tian et al., 2024; Wang et al., 2024). Single-cell lipidomics and spatial lipid imaging are therefore needed to determine whether ferroptosis vulnerability reflects neuron-intrinsic PUFA enrichment, glial lipid remodeling, or both, and to identify cell-type-specific lipid biomarkers in PD (Ma et al., 2024; Cheng et al., 2026; Xiao et al., 2026).

### 4.4 Mitochondrial dysfunction as an amplifier of ferroptotic signaling

Mitochondria serve as both the primary source of reactive oxygen species and the principal target of ferroptotic damage in rotenone-induced neurotoxicity. Rotenone directly inhibits mitochondrial complex I, blocking electron transfer from NADH to ubiquinone and causing electron leakage that generates superoxide radicals (Linjacki et al., 2024; Ma et al., 2024; Lin et al., 2026). The resulting mitochondrial oxidative stress triggers a cascade of deleterious events: decreased mitochondrial membrane potential (ΔΨm), impaired ATP synthesis, and compromised mitochondrial dynamics (Sun et al., 2024; Lin et al., 2026). JC-1 staining reveals significant mitochondrial depolarization in rotenone-treated neurons, consistent with mitochondrial dysfunction (Hu et al., 2023; Sun et al., 2024).

The interplay between mitochondrial dysfunction and iron metabolism creates a self-amplifying cycle of ferroptotic injury. Mitochondrial iron-sulfur cluster-containing proteins, including complex I subunits and aconitase, are exquisitely sensitive to oxidative damage (Linjacki et al., 2024). Rotenone-induced destabilization of these iron-sulfur clusters releases free iron within mitochondria, fueling Fenton chemistry and exacerbating oxidative damage (Linjacki et al., 2024). Additionally, impaired mitophagy, as evidenced by reduced PINK1 and Parkin expression, allows the accumulation of damaged mitochondria that continue to produce reactive oxygen species (Abuelezz et al., 2025; Wang et al., 2026). The failure to eliminate dysfunctional mitochondria contributes to the progressive oxidative stress that drives ferroptotic cell death (Ma et al., 2024; Abuelezz et al., 2025; Wang et al., 2026). This mitochondria-centered ferroptosis mechanism is not restricted to rotenone. Zheng et al., 2026 showed that glucose intolerance synergizes with MPTP to impair mitochondrial respiration, collapse ΔΨm, disrupt cristae, and provoke iron overload, GSH depletion, lipid peroxidation, GPX4 downregulation, and ACSL4 upregulation, thereby establishing mitochondrial dysfunction–ferroptosis as a shared node linking metabolic stress to PD pathology (Zheng et al., 2026).

Mitochondrial dynamics are also disrupted by rotenone exposure, with excessive mitochondrial fission promoting ferroptosis. Dynamin-related protein 1 (DRP1)-mediated mitochondrial fragmentation is elevated in rotenone-treated neurons, and DRP1 inhibition attenuates ferroptotic cell death (Sun et al., 2024). Mitochondrial fragmentation facilitates the release of pro-apoptotic factors and amplifies ROS production, creating a feed-forward loop of mitochondrial damage and ferroptosis propagation (Sun et al., 2024). Transmission electron microscopy confirms the presence of fragmented, swollen mitochondria with disrupted cristae in rotenone-treated neurons, morphological features characteristic of ferroptotic cell death (Sun et al., 2024; Lin et al., 2026).

### 4.5 Neuroinflammation-ferroptosis crosstalk and oxidative stress amplification

Microglial activation and neuroinflammation play a dual role in rotenone-induced neurotoxicity, both contributing to and being amplified by ferroptotic processes. Rotenone exposure activates microglia through complement receptor 3 (CR3) and NADPH oxidase 2 (NOX2) pathways, leading to the production of pro-inflammatory cytokines and reactive oxygen species (Cheng et al., 2026; Xiao et al., 2026). The catalytic subunit gp91phox of NOX2 is significantly upregulated in microglia following rotenone exposure, driving the generation of superoxide and hydrogen peroxide that promote neuronal ferroptosis (Tian et al., 2024; Cheng et al., 2026).

The mechanism by which microglial NOX2 activation promotes neuronal ferroptosis involves both direct oxidative damage and iron dysregulation. Microglial-derived ROS, particularly H_2_O_2_, can penetrate neurons and disrupt iron homeostasis by modulating iron regulatory proteins (Tian et al., 2024; Cheng et al., 2026). Additionally, microglial activation releases TNFα, IL-1β, and other pro-inflammatory cytokines that upregulate DMT1 expression and downregulate FPN1 in neurons, promoting iron accumulation and ferroptosis susceptibility (Ma et al., 2024; Tian et al., 2024). The correlation between microglial activation and neuronal iron deposition has been established through co-localization studies showing iron-positive staining in Iba-1^+^ microglia in the substantia nigra of rotenone-treated mice (Ma et al., 2024). Mechanistically, the directionality should be framed as a two-phase, bidirectional process rather than a single linear cause-effect relationship. In the early phase, non-cell-autonomous microglial activation can precede overt neuronal ferroptosis: rotenone-induced gut dysbiosis and CXCL1 elevation, together with microglial CR3/NOX2 activation, generate extracellular ROS and cytokines (TNF-α, IL-1β) that remodel neuronal iron handling by upregulating DMT1 and downregulating FPN1, thereby sensitizing dopaminergic neurons to ferroptosis (Ma et al., 2024; Wang et al., 2024; Ma et al., 2026). This early microglial contribution is therefore best considered a non-cell-autonomous initiator/amplifier. In the later phase, cell-autonomous ferroptotic machinery—GPX4 inactivation, ACSL4/LPCAT3-dependent PUFA-phospholipid remodeling, NCOA4-mediated ferritinophagy, and mitochondrial iron-sulfur cluster release—drives lethal lipid peroxidation (Sun et al., 2024; Liu et al., 2025). Once neurons undergo ferroptosis, they release DAMPs and oxidized lipids that secondarily activate microglia, closing a feed-forward loop (Tian et al., 2024; Wang et al., 2024). Thus, microglial activation is not simply upstream or downstream; it can initiate neuronal vulnerability early and then be reinforced by neuronal ferroptosis-derived DAMPs. Cell-autonomous and non-cell-autonomous mechanisms should be experimentally dissected using microglia-neuron co-cultures, conditional CR3/NOX2 or NCOA4 knockout, and neuron-specific GPX4/ACSL4 manipulations.

The reciprocal relationship between neuroinflammation and ferroptosis creates a feed-forward amplification loop. Ferroptotic neurons release damage-associated molecular patterns (DAMPs), including lipid peroxidation products and oxidized DNA, which activate microglia and propagate inflammatory responses (Ma et al., 2024; Tian et al., 2024). This neuroinflammation-ferroptosis crosstalk is particularly relevant to the progressive nature of PD, as the vicious cycle of microglial activation, neuronal iron accumulation, and ferroptotic death drives continuous neurodegeneration (Tian et al., 2024; Cheng et al., 2026). Pharmacological inhibition of microglial gp91phox using AAV-mediated knockdown or genetic deletion attenuates rotenone-induced ferroptosis and cognitive deficits, highlighting the therapeutic potential of targeting this pathway (Tian et al., 2024).

### 4.6 SIRT1-Nrf2 signaling and its role in ferroptosis regulation

The SIRT1-Nrf2 signaling axis constitutes a critical protective mechanism against ferroptosis, and rotenone profoundly disrupts this pathway. SIRT1, an NAD^+^-dependent deacetylase, promotes the nuclear translocation and transcriptional activity of Nrf2, which in turn regulates the expression of antioxidant and anti-ferroptotic genes (Abd El-Khalik et al., 2025; Ma et al., 2026). Rotenone exposure significantly decreases SIRT1 and Nrf2 expression in neurons, with the reduction occurring through both ubiquitin-proteasome and autolysosomal degradation pathways (Ma et al., 2026). The consequence is impaired transcription of Nrf2 target genes, including GPX4, SLC7A11, FTH1, and heme oxygenase-1 (HO-1), compromising cellular antioxidant capacity (Abd El-Khalik et al., 2025; Ma et al., 2026).

The restoration of SIRT1-Nrf2 signaling by pharmacological activators confirms its protective role against rotenone-induced ferroptosis. Resveratrol, a SIRT1 activator, and tert-butylhydroquinone (TBHQ), an Nrf2 activator, both attenuate rotenone-induced ferroptosis *in vitro* and *in vivo* (Abd El-Khalik et al., 2025; Ma et al., 2026). These agents restore GPX4 expression, reduce lipid peroxidation, and preserve mitochondrial function, ultimately protecting dopaminergic neurons from ferroptotic death (Abd El-Khalik et al., 2025; Ma et al., 2026). The downstream mechanisms involve upregulation of ferroportin 1 (FPN1), promoting iron export and reducing cellular iron accumulation, as well as GPX4 induction, enhancing lipid peroxide clearance (Ma et al., 2026). Notably, the ferroptosis inhibitors Fer-1 and DFO also reverse rotenone-induced SIRT1-Nrf2 suppression, suggesting a reciprocal relationship between ferroptosis and Nrf2 pathway inhibition (Abd El-Khalik et al., 2025).

The interaction between circadian rhythm regulation and ferroptosis adds another layer of complexity to rotenone-induced neurotoxicity. Rotenone exposure suppresses Bmal1 expression, a core circadian clock gene, and Bmal1 normally promotes Nrf2 transcriptional activity (Shuai et al., 2026). Nobiletin, a polymethoxylated flavone that activates Bmal1 expression, protects against rotenone-induced ferroptosis through Bmal1/Nrf2 pathway activation (Shuai et al., 2026). This finding suggests that circadian disruption, a common feature of PD, may contribute to ferroptosis susceptibility by impairing the rhythmic activation of antioxidant defenses (Shuai et al., 2026). This circadian–redox coupling extends beyond rotenone. Glucose-responsive AMPK–TET2–5hmc oscillation links metabolic stress to circadian transcription and PD relevance (Chu et al., 2026, npj Parkinson’s Disease), placing glycemic fluctuations upstream of Bmal1/Nrf2 and SIRT1–Nrf2 and broadening the mechanistic framework beyond rotenone alone (Chu et al., 2026).

### 4.7 Apoptosis-ferroptosis cross-talk in rotenone-induced neurotoxicity

The relationship between apoptosis and ferroptosis in rotenone-induced cell death involves complex cross-talk mechanisms. Rotenone simultaneously activates both apoptotic and ferroptotic pathways, with the relative contribution of each death modality depending on the cellular context and exposure conditions (Li et al., 2025). The apoptotic pathway is characterized by mitochondrial membrane potential loss, Bax upregulation, Bcl-2 downregulation, and caspase-3 activation, while the ferroptotic pathway involves iron accumulation, GPX4 suppression, and lipid peroxidation (Li et al., 2025).

Mechanistically, rotenone-induced ROS activate BAX, which oligomerizes at the mitochondrial outer membrane, promotes cytochrome c release, and triggers caspase-9/-3 activation (Li et al., 2025). BAX-dependent mitochondrial permeabilization also liberates mitochondrial iron, thereby expanding the labile Fe^2+^ pool and feeding lipid peroxidation (Liu et al., 2025). Concurrently, lipid peroxidation products such as 4-HNE and MDA, together with oxidized phospholipids including 15-HpETE-PE and 17-HpDTE-PE, modify mitochondrial proteins and promote mPT opening; mPT collapse of ΔΨm amplifies ROS, ATP depletion, cytochrome c release, and further iron release, thereby integrating apoptotic and ferroptotic execution (Sun et al., 2021; von Krusenstiern et al., 2023). Caspase activation and ferroptosis are therefore not simply parallel; ferroptotic signaling can promote caspase-3 activation, as Fer-1 and DFO reduce rotenone-induced caspase-3 cleavage, whereas caspase inhibition alone does not fully rescue rotenone-treated neurons (Sun et al., 2024; Li et al., 2025). Microglial NOX2-derived ROS can further sensitize neurons to this BAX/mPT-dependent crosstalk (Wang et al., 2024). This bidirectional interaction suggests that single-pathway blockade may be insufficient. Combinatorial targeting of apoptosis and ferroptosis is likely to be beneficial in severe or early rotenone injury, where BAX/mPT-dependent apoptosis and iron-dependent lipid peroxidation coexist; however, chronic broad inhibition of both pathways may be potentially toxic by suppressing physiological apoptosis and ferroptosis involved in immune surveillance, development, and tumor suppression (Dixon et al., 2012; Stockwell, 2022). Thus, combinatorial strategies should be mechanism-guided, time-limited, and biomarker-monitored—for example, using BAX/mPT inhibitors together with ferroptosis inhibitors only when both caspase activation and lipid peroxidation are elevated—rather than applied as indiscriminate dual blockade (Mansour et al., 2023b; Li et al., 2025). Rotenone-induced mitochondrial ROS production triggers the intrinsic apoptotic pathway through mitochondrial membrane permeabilization and cytochrome c release (Li et al., 2025). Simultaneously, ROS-mediated iron release and lipid peroxidation activate the ferroptotic pathway (Li et al., 2025). The interplay between these pathways is evidenced by the observation that ferroptosis inhibitors (Fer-1, DFO) reduce rotenone-induced caspase-3 activation, suggesting that ferroptotic signaling can promote apoptotic execution. Conversely, apoptosis inhibition does not fully protect against rotenone-induced cell death, indicating that ferroptosis operates as a parallel death pathway (Li et al., 2025).

The molecular mechanism of apoptosis-ferroptosis cross-talk involves BAX-dependent mitochondrial permeabilization. BAX, the pro-apoptotic Bcl-2 family member, can also contribute to ferroptosis by promoting mitochondrial iron release (Liu et al., 2025). Conversely, ferroptotic lipid peroxidation products can activate the mitochondrial permeability transition pore, triggering apoptosis (Liu et al., 2025). The coordinated activation of both death pathways ensures the elimination of severely damaged neurons, but also complicates therapeutic strategies targeting a single death modality (Mansour et al., 2023a; Li et al., 2025).

### 4.8 Limitations of the rotenone model and generalizability of ferroptosis mechanisms to genetic and idiopathic PD

Rotenone remains one of the most instructive toxin-based models of PD because chronic systemic exposure reproduces selective nigrostriatal dopaminergic degeneration, α-synuclein/ubiquitin-positive inclusions, motor deficits, and mitochondrial complex I inhibition (Betarbet et al., 2000; Cannon et al., 2009). These features have made it indispensable for mechanistic studies of environmental toxin-associated parkinsonism and for preclinical testing of ferroptosis inhibitors (Radad et al., 2019; Ibarra-Gutiérrez et al., 2023). Nevertheless, its validity is conditional rather than absolute. The model is highly sensitive to dose, route, formulation, duration, species, strain, age, sex, and husbandry conditions (Cannon et al., 2009; Radad et al., 2019; Lal et al., 2024). Chronic low-dose systemic administration—most commonly intraperitoneal, subcutaneous, intravenous, or intragastric—can produce relatively selective nigrostriatal injury, whereas acute or high-dose exposure often causes severe systemic toxicity, including weight loss, hepatic and cardiac injury, gastrointestinal dysfunction, and mortality, which can confound neuroprotection readouts (Cannon et al., 2009; Radad et al., 2019; Amaral et al., 2025). Species differences are also substantial: rats, particularly certain strains, are generally more susceptible to rotenone-induced nigrostriatal degeneration than mice, and pharmacokinetic differences alter brain exposure and lesion variability (Cannon et al., 2009; Radad et al., 2019; Ma et al., 2024). Because rotenone is lipophilic and crosses the blood-brain barrier, peripheral toxicity and central effects are difficult to dissociate completel (Radad et al., 2019; Ma et al., 2024). Moreover, rotenone is not a model of idiopathic PD; it primarily models mitochondrial complex I inhibition and the downstream oxidative/ferroptotic cascade, rather than the aging-dependent, multifactorial, and genetically heterogeneous pathogenesis of idiopathic PD (Kalia and Lang, 2015; Poewe et al., 2017; Ibarra-Gutiérrez et al., 2023). It does not fully recapitulate LRRK2, GBA, SNCA, PARKIN, or PINK1 lesions, progressive α-synuclein propagation, or the full spectrum of non-motor pathology (Kalia and Lang, 2015; Mansour et al., 2023a; Abuelezz et al., 2025).

These limitations do not invalidate the ferroptosis findings, but they define their translational scope. The core ferroptotic nodes identified in rotenone models—iron dyshomeostasis with DMT1 upregulation and FPN1 downregulation, labile Fe^2+^ accumulation, Fenton-driven lipid peroxidation, Xc^−^–GSH–GPX4 axis inactivation, ACSL4/LPCAT3-mediated PUFA phospholipid remodeling, 15-LOX/PEBP1-derived death signals, mitochondrial ROS amplification, NCOA4-dependent ferritinophagy, and microglial CR3/NOX2-driven neuroinflammation—are biologically plausible in idiopathic PD and in several genetic forms of PD, but the strength of evidence differs across nodes (Ma et al., 2024; Sun et al., 2024; Tian et al., 2024; Wang et al., 2024; Liu et al., 2025). For idiopathic PD, postmortem and imaging studies have long linked nigral iron accumulation, oxidative stress, and lipid peroxidation to dopaminergic vulnerability, and recent work supports GPX4 loss, ferroptosis-related transcriptional signatures, and iron-dependent mitochondrial injury as contributors (Hu et al., 2023; Ma et al., 2024). Nevertheless, human idiopathic PD is not a single ferroptosis disease; ferroptosis likely interacts with α-synuclein proteostasis, lysosomal dysfunction, neuroinflammation, and aging-related iron retention, and its relative contribution may vary among patients (Poewe et al., 2017; Li et al., 2025). For genetic PD, the translation is also pathway-dependent. PARKIN/PINK1 loss impairs mitophagy and mitochondrial quality control, which can increase labile iron, mitochondrial ROS, and susceptibility to lipid peroxidation; this provides a mechanistic bridge between genetic PD and ferroptosis (Abuelezz et al., 2025). LRRK2 and α-synuclein pathology may further amplify iron dyshomeostasis and lipid peroxidation, and HSP90/CDC37 inhibition has been shown to modulate both ferroptosis and necroptosis while affecting PD-relevant proteins such as LRRK2 and α-synuclein (Mansour et al., 2023b; Abuelezz et al., 2025). However, rotenone does not directly model these primary genetic lesions, and ferroptosis inhibitors in rotenone models cannot be assumed to rescue genetic PD without testing in LRRK2, GBA, SNCA, PARKIN, or PINK1 models (Thirugnanam and Santhakumar, 2022; Mansour et al., 2023a; Abuelezz et al., 2025). Similarly, rotenone models complex I inhibition and oxidative stress more faithfully than they model α-synuclein seeding, propagation, and templating, which are central to idiopathic and SNCA-linked PD (Kalia and Lang, 2015). The gut microbiota-CXCL1-PPARγ axis and microglial gp91phox/CR3-NOX2 pathways identified in rotenone models may be more generalizable because neuroinflammation and gut-brain interactions are relevant across PD subtypes, but this remains to be confirmed in genetic and idiopathic models (Ma et al., 2024; Tian et al., 2024; Wang et al., 2024).

For translational credibility, several caveats should be made explicit. First, dose and route must be reported rigorously, and the lowest effective dose that produces nigrostriatal injury without overt systemic toxicity should be preferred; otherwise, nonspecific toxicity may be misinterpreted as ferroptosis-specific neuroprotection (Cannon et al., 2009; Radad et al., 2019; Amaral et al., 2025). Second, both sexes and multiple species should be included, because sex hormones, iron metabolism, and lipid peroxidation differ across sexes and strains (Radad et al., 2019; Liang et al., 2023). Third, target engagement and pharmacodynamic biomarkers—such as 4-HNE, MDA, oxidized phospholipids, ferritin, GPX4 activity, and labile iron—should be measured in brain and biofluids to confirm that protection is mediated by ferroptosis inhibition rather than systemic effects (Hirata et al., 2022; Hu et al., 2023; Liu et al., 2025). Fourth, rotenone findings should be validated in genetic PD models (e.g., PINK1^−/−^, Parkin^−/−^, LRRK2 mutant, GBA mutant, and α-synuclein preformed fibril models) and in human iPSC-derived dopaminergic neurons and organoids, where possible (Thirugnanam and Santhakumar, 2022; Mansour et al., 2023a; Abuelezz et al., 2025). Fifth, because idiopathic PD is heterogeneous, ferroptosis-targeted therapies may benefit only a subgroup—perhaps those with iron overload, mitochondrial dysfunction, or specific lipid peroxidation signatures—so biomarker-based patient stratification will be essential. Finally, rotenone is best regarded as a mechanistic model of complex I inhibition–driven ferroptosis that can generate testable hypotheses, not as a surrogate for all PD; conclusions about disease modification should be tempered accordingly and confirmed across complementary models (Kalia and Lang, 2015; Kanthasamy et al., 2019; Ibarra-Gutiérrez et al., 2023).

## 5 Mechanistic hierarchy and temporal sequence of rotenone-induced ferroptosis

To clarify the mechanistic hierarchy and causal sequence, rotenone-induced ferroptosis should be conceptualized as a three-tier cascade rather than a single linear pathway. The initiating trigger is mitochondrial complex I inhibition. Rotenone directly binds and inhibits NADH:ubiquinone oxidoreductase, blocking electron transfer from NADH to ubiquinone and causing electron leakage at the inner mitochondrial membran (Ibarra-Gutiérrez et al., 2023). This proximal event occurs within minutes to hours of exposure and is the primary upstream cause of subsequent pathology. The rotenone model reproduces selective nigrostriatal dopaminergic degeneration, α-synuclein-containing inclusions, and motor deficits, establishing the pathological relevance of this initiating insult (Betarbet et al., 2000; Cannon et al., 2009). At this earliest stage, ferroptosis is not yet the primary driver; rather, mitochondrial electron transport chain inhibition and the resulting bioenergetic failure and reactive oxygen species (ROS) production set the stage for downstream death signaling.

The early upstream amplification tier develops over hours to days *in vitro* and over days to approximately 2 weeks *in vivo*. During this phase, mitochondrial ROS overproduction, loss of mitochondrial membrane potential, impaired ATP synthesis, and destabilization of iron–sulfur clusters release free iron into the mitochondrial matrix (Gao et al., 2019; Lin et al., 2026). In parallel, rotenone suppresses the SIRT1–Nrf2 axis, reducing the transcriptional capacity to maintain antioxidant and iron-handling genes (Liu et al., 2025; Ma et al., 2026). This suppression contributes to downregulation of xCT (SLC7A11), glutathione depletion, and GPX4 inactivation, thereby compromising the primary defense against lipid peroxidation (Yang et al., 2014; Hu et al., 2023; Sun et al., 2024). Rotenone also disrupts iron transport by upregulating DMT1 and downregulating FPN1, while iron regulatory proteins promote iron retention (Ma et al., 2024; Sun et al., 2024; Liu et al., 2025). In addition, NCOA4-mediated ferritinophagy degrades ferritin and liberates stored iron (Sun et al., 2024). Lipid metabolic remodeling further lowers the threshold for ferroptosis: ACSL4 and LPCAT3 enrich membrane phospholipids with oxidation-prone polyunsaturated fatty acid (PUFA) substrates (Doll et al., 2017), and the 15-LOX/PEBP1 complex generates pro-ferroptotic oxidized phospholipid signals such as 15-HpETE-PE and 17-HpDTE-PE (Kagan et al., 2017; Sun et al., 2021). Mitochondrial fission mediated by DRP1 and impaired mitophagy amplify ROS production and mitochondrial damage (Sun et al., 2024; Sun et al., 2024). Notably, *in vivo* studies show that intragastric rotenone can induce iron deposition in the substantia nigra and striatum as early as 2 weeks after exposure, before overt dopaminergic neuron loss (Ma et al., 2024). Early neuroinflammation, including gut microbiota–CXCL1-driven microglial activation in the substantia nigra, can also occur during this phase and further sensitize neurons to iron-dependent injury (Ma et al., 2024). These events are not strictly sequential; they operate in parallel and reinforce one another through reciprocal feedback.

The terminal execution tier is defined by labile ferrous iron–driven Fenton chemistry and lethal lipid peroxidation. Labile Fe^2+^ reacts with hydrogen peroxide to generate hydroxyl radicals, which initiate peroxidative chain reactions in PUFA-containing membrane phospholipids (Dixon et al., 2012; Yan et al., 2021). The accumulation of 15-HpETE-PE and 17-HpDTE-PE, together with failure of iPLA2β-mediated clearance of oxidized phospholipids, commits cells to ferroptotic death (Kagan et al., 2017; Sun et al., 2021). When approximately 2% of membrane lipids become peroxidized, membrane integrity is irreversibly compromised and rupture occurs (von Krusenstiern et al., 2023). Reactive aldehydes such as 4-hydroxynonenal and malondialdehyde form adducts with proteins, lipids, and DNA, amplifying cellular damage (Hu et al., 2023; Tian et al., 2024). GPX4 loss removes the final safeguard against phospholipid hydroperoxide accumulation (Yang et al., 2014). At this stage, ferroptosis functions as the terminal executioner of dopaminergic neuron death. However, because iron deposition and lipid peroxidation precede overt neuronal loss in rotenone models, ferroptosis is not merely a downstream epiphenomenon; once engaged, it becomes a driver of degeneration.

The causal sequence can therefore be summarized as follows: rotenone exposure leads to mitochondrial complex I inhibition, which triggers mitochondrial ROS overproduction and bioenergetic failure; this is followed by SIRT1–Nrf2 suppression, System Xc^−^–GPX4 axis failure, iron transport dysregulation, NCOA4-mediated ferritinophagy, and PUFA-phospholipid remodeling; these early amplifiers converge on labile Fe^2+^ accumulation and Fenton-driven lipid peroxidation; ferroptosis then executes membrane rupture and neuronal death; and ferroptotic neurons release damage-associated molecular patterns that activate microglial CR3/NOX2 signaling and pro-inflammatory cytokines such as TNFα and IL-1β, which further disrupt neuronal iron homeostasis and create a feed-forward amplification loop (Tian et al., 2024; Wang et al., 2024). Ferritinophagy-mediated iron release and neuroinflammation thereby propagate ferroptosis beyond the initial insult (Sun et al., 2024; Tian et al., 2024; Wang et al., 2024). Apoptosis–ferroptosis crosstalk, involving ROS, BAX-dependent mitochondrial permeabilization, and cytochrome c release, further links ferroptosis to parallel death pathways and complicates single-target therapeutic strategies (Li et al., 2025). This hierarchical framework indicates that ferroptosis is an early amplifying and terminal execution mechanism rather than the primary initiating event, but it also explains why ferroptosis inhibition can be disease-modifying once iron-dependent lipid peroxidation has been engaged. It further suggests that therapeutic windows may differ: targeting complex I, mitochondrial ROS, or SIRT1–Nrf2 early may prevent ferroptosis initiation, whereas targeting labile iron, lipid radicals, ferritinophagy, or neuroinflammatory amplification later may slow or reverse progressive dopaminergic degeneration (Stockwell, 2022; Wang et al., 2023a).

## 6 Therapeutic implications of targeting ferroptosis in rotenone-induced PD

Accumulating evidence from the reviewed studies demonstrates that pharmacological modulation of ferroptosis—via diverse molecular targets—confers significant neuroprotection in rotenone-induced PD models. These findings collectively supports ferroptosis as a candidate pathogenic contributor in rotenone-induced Parkinsonian models, while its centrality in human PD remains to be established and highlight several classes of compounds with distinct but converging mechanisms of action. The major therapeutic strategies and their mechanistic underpinnings are summarized below in Figure 3 and Table 1. Critically, the compounds listed in Table 1 and Figure 4 are not mechanistically equivalent with respect to ferroptosis dependence. Fer-1, liproxstatin-1, DFO, deferiprone, and CNO target core ferroptotic nodes—lipid radical trapping, labile iron, or NCOA4-mediated ferritinophagy—and their protection is therefore most directly attributable to ferroptosis inhibition (Hirata et al., 2022; Sun et al., 2024; Tian et al., 2024). Carvedilol, resveratrol, TBHQ, NBP, and idebenone restore Nrf2/GPX4/xCT or mitochondrial antioxidant capacity, but they also modulate PINK1/Parkin mitophagy, mitochondrial function, and neuroinflammation, so their effects are ferroptosis-associated rather than strictly ferroptosis-specific (Avcı et al., 2020; Hu et al., 2023; Abuelezz et al., 2025; Liu et al., 2025; Ma et al., 2026). Lapatinib and pazopanib inhibit HSP90/CDC37 and kinase cascades and concurrently suppress necroptosis and apoptosis, indicating that their neuroprotection is multi-pathway and not exclusively ferroptosis-dependent (Mansour et al., 2023a; Mansour et al., 2023b). The gut microbiota–CXCL1–PPARγ intervention similarly acts upstream of both ferroptosis and microglial inflammation (Ma et al., 2024). Therefore, future studies should establish specificity by testing whether protection is reversed by ferroptosis inducers (e.g., RSL3 or erastin), whether ferroptotic markers (4-HNE, MDA, oxidized phospholipids, GPX4 activity, labile Fe^2+^) are normalized, and whether caspase/RIPK/MLKL or mitophagy readouts are unaffected or separately modulated (Dixon et al., 2012; Yang et al., 2014; Mansour et al., 2023a; Mansour et al., 2023b; Sun et al., 2024; Liu et al., 2025).

**FIGURE 3:**
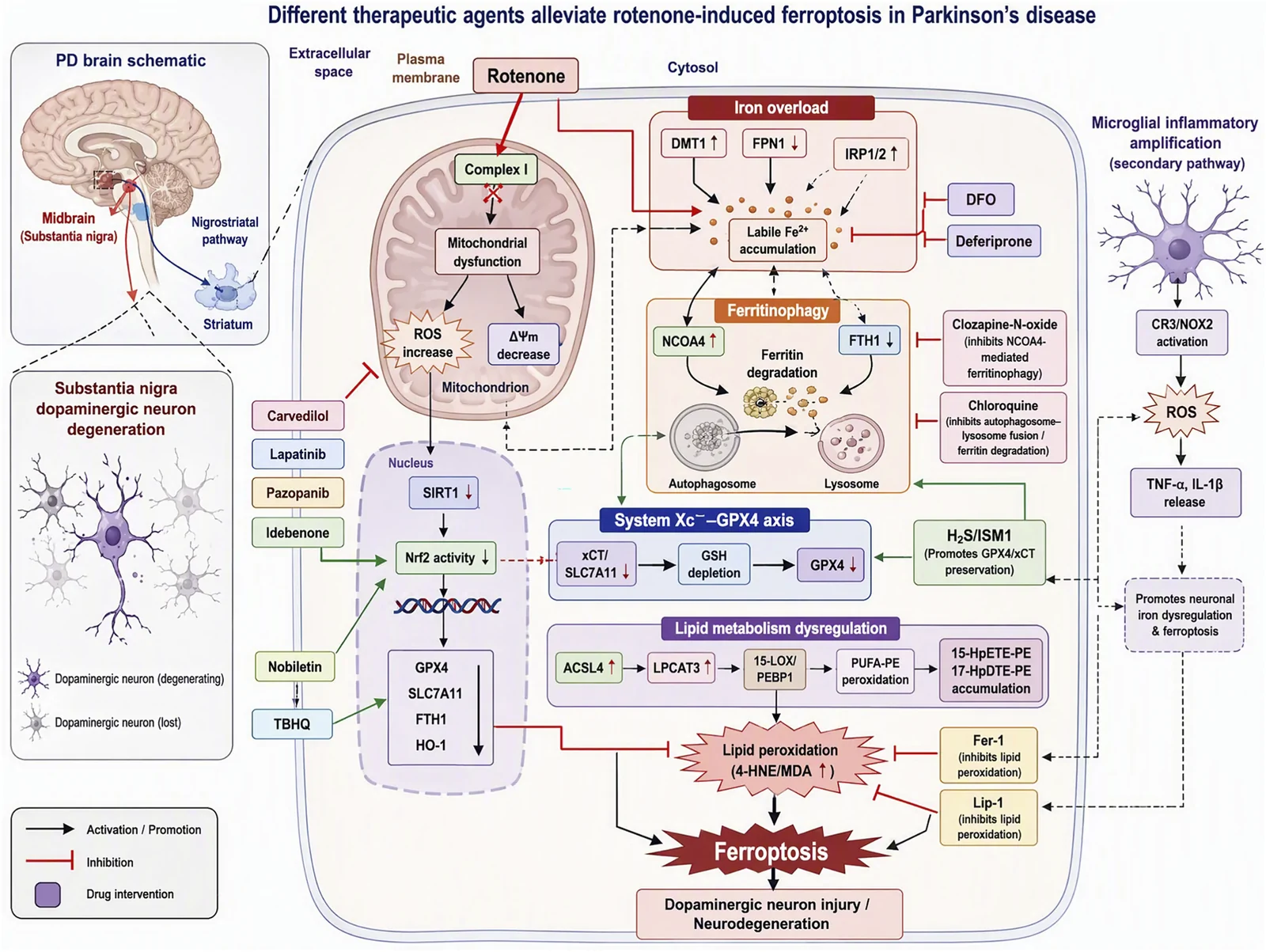
Therapeutic targeting of rotenone-induced ferroptosis. Schematic overview of pharmacological strategies that attenuate rotenone-induced dopaminergic neurodegeneration by modulating ferroptosis. Agents are grouped by primary mechanism: direct ferroptosis inhibitors (Fer-1, liproxstatin-1), iron chelators (DFO, deferiprone), ferritinophagy inhibitors (CNO), Nrf2/GPX4 activators (carvedilol, resveratrol, TBHQ, NBP, idebenone, H_2_S donors), lipid peroxidation and mitochondrial modulators (GIF compounds), multi-target kinase/HSP90 inhibitors (lapatinib, pazopanib), and microbiota–PPARγ modulators (linoleic acid). Solid arrows denote direct or established ferroptosis-dependent protection; dashed arrows denote ferroptosis-associated or multi-pathway effects involving mitophagy, apoptosis, necroptosis, and neuroinflammation. The figure corresponds to the agents and mechanisms summarized in Table 1.

**TABLE 1:** Pharmacological mechanisms of drugs inhibiting rotenone-mediated ferroptosis in Parkinson’s disease.

Agent	Species/model	Dose, route, duration	Behavioral outcomes	BBB penetration	Key pharmacological/mechanistic effects	References
Carvedilol	Male Wistar rats; rotenone 1.5 mg/kg s.c. every other day × 21 days	10 mg/kg/day, p.o., × 25 days (started 4 days before rotenone)	Improved open-field distance, speed, rearing, immobility; improved rotarod fall latency and grip strength	Yes; lipophilic, crosses BBB	↑ Nrf2, GPX4, PINK1, PARKIN, catalase; ↓ ACSL4, NF-κB, MDA; ↓ α-syn; ↑ TH; preserved mitochondrial/mitophagy balance	Cheng et al. (2026)
NaHS (H_2_S donor)	HT22 hippocampal cells; Sprague-Dawley rats (hippocampus)	*In vitro*: 0.1, 0.3, 1 mM, 30 min pretreatment. *In vivo*: 30 or 100 μmol/kg i.p. daily × 6 weeks; rotenone 2 mg/kg s.c. × 5 weeks	NR in these papers	H_2_S is gaseous; likely crosses BBB	Inhibits ferroptosis and ferritinophagy; ↑ GPX4, FTH1, GSH; ↓ Fe^2+^, MDA, lipid ROS, ACSL4, NCOA4, LC3-II/I; preserves mitochondrial morphology	Linjacki et al. (2024), Chen et al. (2025), Shuai et al. (2026)
Resveratrol	C57BL/6 mice; rotenone 1.5 mg/kg i.p. × 21 days	20 mg/kg/day i.p. (Liu et al., 2025) or 30 mg/kg i.p. (Ma et al., 2026), × 21 days	Improved gait (Liu et al., 2025); improved Morris water maze (Ma et al., 2026)	Yes; brain-penetrant	Activates SIRT1/Nrf2; ↑ Fpn1, GPX4, GSH; ↓ iron, MDA, ACSL4, PTGS2; inhibits ferroptosis; in Ma et al., suppresses microglial activation, synaptic pruning, neuroinflammation	Liu et al. (2025), Ma et al. (2026)
TBHQ	C57BL/6 mice; rotenone 1.5 mg/kg i.p. × 21 days	50 mg/kg/day i.p. (Liu et al., 2025) or 25 mg/kg i.p. (Ma et al., 2026)	Improved gait/morris water maze	Likely crosses BBB; small Nrf2 activator	Activates Nrf2; ↑ GPX4, GSH; ↓ ferroptosis, oxidative stress, inflammation; similar to resveratrol	Liu et al. (2025), Ma et al. (2026)
Deferoxamine (DFO)	MELAS fibroblasts, SH-SY5Y cells, mice	*In vitro*: 1–100 μM. *In vivo*: 100 mg/kg gavage (Tian et al.); rotenone 1.5 mg/kg i.p. × 21 days in mice	NR for behavior in most studies	Limited; hydrophilic, poor BBB penetration	Iron chelation; ↓ Fe^2+^, lipid peroxidation, ferroptosis; restores mitochondrial morphology; *in vivo* ↓ MDA, GSSG/GSH	Sun et al. (2024), Tian et al. (2024)
Idebenone	Male Sprague-Dawley rats; rotenone 5 μg intranigral	200 mg/kg, p.o., × 28 days	Improved open-field, rotarod, cylinder test	Yes; lipophilic, crosses BBB	↑ GPx-4, NQO1; ↓ MDA, lipid peroxidation; protects TH^+^ neurons; reduces ferroptosis markers	Avcı et al. (2020)
Lapatinib ditosylate	Male Wistar rats; rotenone 2 mg/kg s.c. × 3 weeks	100 mg/kg, p.o., daily × 3 weeks	Improved open-field, rotarod, grip strength	Yes; used for brain metastases	Inhibits p-EGFR/PLCγ/PKCβII/ACSL4; inhibits HSP90/CDC37; ↑ GPX4/GSH; ↓ iron, TfR1, 4-HNE, PTGS2, α-syn, LRRK2, c-ABL; ↑ TH, DA	Mansour et al. (2023a)
Pazopanib	Male Wistar rats; rotenone 2 mg/kg s.c. × 3 weeks	15 mg/kg, p.o., daily × 3 weeks	Improved open-field, rotarod, grip strength	Yes; small molecule penetrates BBB	Inhibits necroptosis (p-RIPK1/p-RIPK3/p-MLKL) and ferroptosis (p-VEGFR2/PLCγ/PKCβII/ACSL4); ↑ GPX4/GSH; ↓ iron, 4-HNE, PTGS2; inhibits HSP90/CDC37; ↓ α-syn, LRRK2, c-ABL; ↑ DA, TH	Mansour et al. (2023b)
Clozapine-N-oxide (CNO)	C57BL/6 mice; rotenone 1.5 mg/kg i.p. × 3 weeks	1 mg/kg s.c. twice daily; CLZ and NDC same dose	Improved gait and rotarod	Clozapine crosses BBB; CNO likely	Inhibits NCOA4-mediated ferritinophagy and ferroptosis; ↓ Fe^2+^, MDA, lipid peroxidation, ACSL4, COX2; ↑ GPX4, ferritin, P62; no leukopenia vs. CLZ	Sun et al. (2024)
DL-3-n-butylphthalide (NBP)	Male Sprague-Dawley rats; rotenone 2.5 mg/kg i.p. × 28 days	80 mg/kg, i.g., from day 14–28	Improved rotarod, balance beam, foot fault	Yes; crosses BBB	↓ iron deposition, MDA, oxidative stress; ↑ GSH, xCT, GPX4; ↓ α-syn; protects mitochondrial structure; ↑ TH	Hu et al. (2023)
N-Acetylcysteine (NAC)	SH-SY5Y cells; rotenone 0.01–1 μM	5, 10 mM; 1 h pretreatment	NR	Yes; crosses BBB	ROS scavenger; ↓ ROS, Nrf2/HO-1, NCOA4, caspase-3, ATG5, p62; ↑ GPX4; inhibits ferroptosis, autophagy, apoptosis	Chen et al. (2026)
Ferrostatin-1/liproxstatin-1	SH-SY5Y, HT22 cells; mice	Fer-1: 5 μM *in vitro*. Lipo-1: 10 mg/kg i.p. in mice; rotenone 1.5 mg/kg i.p. × 21 days	Lipo-1 improved Morris water maze	Lipo-1 brain-penetrant; Fer-1 limited	Inhibit lipid peroxidation/ferroptosis; ↓ Fe^2+^, MDA, ACSL4, COX2; ↑ GPX4; *in vivo* reduced neurodegeneration and cognitive deficits	Hirata et al. (2022); Tian et al. (2024)
Linoleic acid	Aged C57BL/6 mice; CXCL1 i.v. model	60 mg/kg/day, i.g., 1 week pre + 2 weeks	Improved pole test	Yes; fatty acid crosses BBB	PPARγ activation; ↓ microglial activation, iron deposition, dopaminergic loss; ↑ PPARγ; restored DA, DOPAC, HVA	Ma et al. (2024)
GIF-2165X-G1	Rotenone-treated HT22 cells	1 μM, *In vitro*, added to culture medium	*In vitro*, added to culture medium	​	Fully blocked 10 mM glutamate- and 1 µM erastin-induced loss of viability at 24 h. Reduced erastin-induced mitochondrial ROS at 8 h. Did not reverse 5 µM rotenone-induced loss of viability at 24 h, MMP depolarization, nuclear fragmentation, or mitochondrial ROS. Prevented delayed rotenone-induced LDH release at 48 h but did not restore viability.	Hirata et al. (2022)
GIF-2014	Rotenone-treated HT22 cells	0.1 µM, *In vitro*, added to culture medium	*In vitro*, added to culture medium	​	Similar to GIF-2165X-G1: fully blocked glutamate- and erastin-induced viability loss; reduced erastin-induced ROS; did not protect against rotenone-induced apoptosis, MMP depolarization, nuclear fragmentation, or ROS; prevented delayed rotenone-induced LDH release at 48 h.	Hirata et al. (2022)
GIF-2115	Rotenone-treated HT22 cells	0.1 µM, *In vitro*, added to culture medium	*In vitro*, added to culture medium	​	Similar protective profile: blocked glutamate/erastin toxicity and erastin-induced ROS; no protection against rotenone-induced apoptosis, MMP depolarization, nuclear fragmentation, or ROS; prevented delayed rotenone-induced LDH release at 48 h.	Hirata et al. (2022)
PLX5622	12-month-old C57BL/6 mice; rotenone 6.25 mg/kg oral, 5 days/week	1,200 ppm in diet	NR for behavior in excerpt	Yes; brain-penetrant CSF1R inhibitor	Microglia depletion; blocked iron deposition, ferroptosis (GPX4), dopaminergic neuron loss	Ma et al. (2024)

**FIGURE 4:**
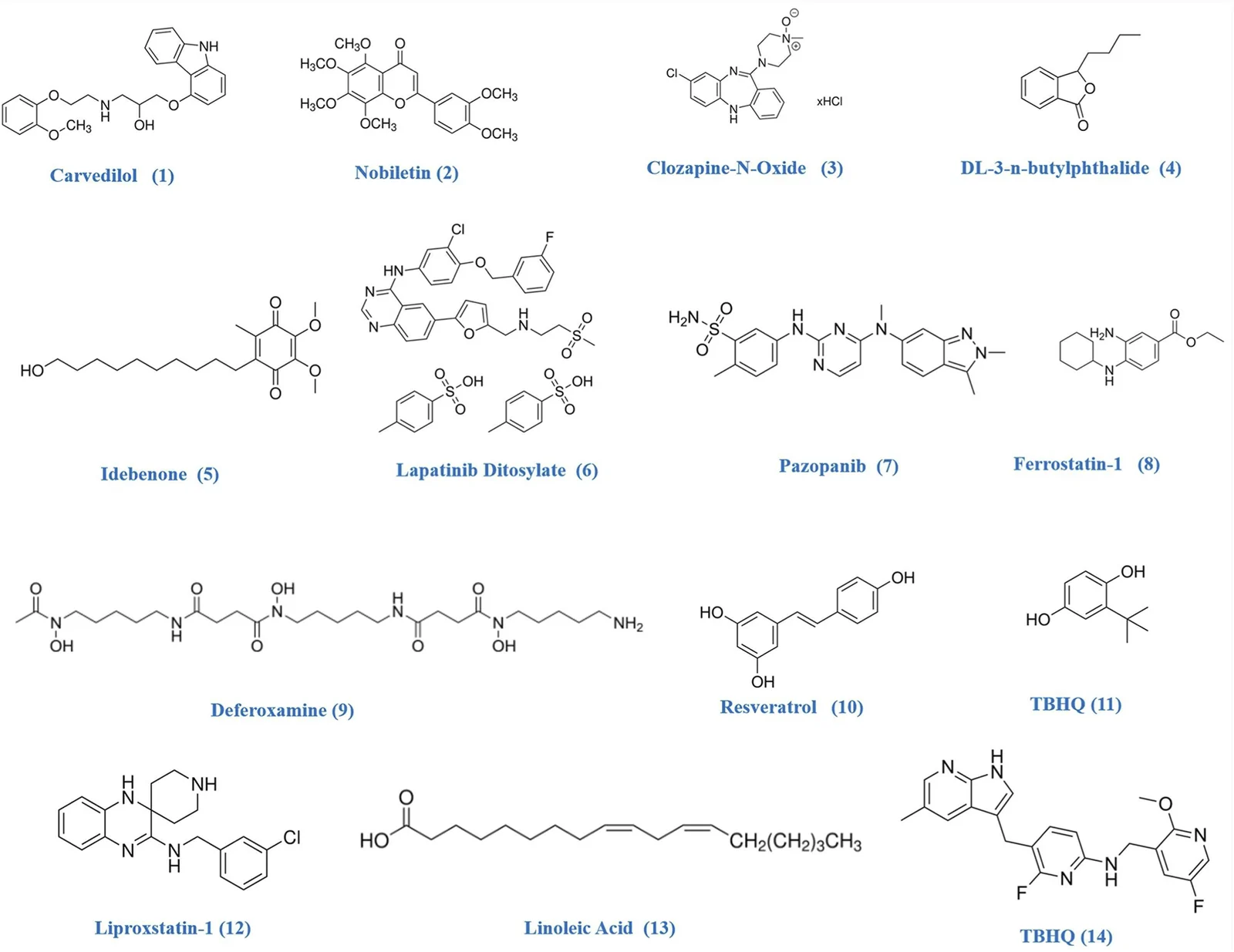
Chemical structures of small-molecule inhibitors of rotenone-induced ferroptosis, showcasing a series of promising lead compounds with potent anti-ferroptotic activity for therapeutic development.

### 6.1 Direct ferroptosis inhibitors

The most straightforward approach involves the use of specific ferroptosis inhibitors. Ferrostatin-1 (Fer-1) and liproxstatin-1 (Lipo-1), both radical-trapping antioxidants, have been shown to effectively block lipid peroxidation and iron-dependent cell death. In rotenone-exposed SH-SY5Y cells and mouse models, Fer-1 and Lipo-1 attenuated mitochondrial damage, reduced iron accumulation and lipid peroxidation, restored GSH levels and GPX4 expression, and improved both motor and cognitive deficits (Sun et al., 2024; Tian et al., 2024) (Figure 3; Table 1). These effects were associated with preservation of dopaminergic neurons and synaptic integrity in the substantia nigra and hippocampus (Ma et al., 2026). Similarly, the iron chelator deferoxamine (DFO) and deferiprone mitigated rotenone-induced ferroptosis by reducing labile iron pools, thereby limiting Fenton chemistry and subsequent lipid peroxidation (Sun et al., 2024; Liu et al., 2025).

### 6.2 Modulators of iron homeostasis and ferritinophagy

Several agents exert anti-ferroptotic effects by regulating iron metabolism and ferritinophagy—the selective autophagic degradation of ferritin. Clozapine-N-oxide (CNO), a metabolite of the atypical antipsychotic clozapine, was found to protect dopaminergic neurons by inhibiting NCOA4-mediated ferritinophagy, thereby preventing ferritin degradation and excessive iron release (Sun et al., 2024). This mechanism was shown to be independent of general autophagy inhibition, as CNO did not affect mitophagy. Importantly, CNO lacked the hematological toxicity associated with its parent compound, highlighting its translational potential (Sun et al., 2024). Nobiletin, a citrus flavonoid, similarly attenuated rotenone-induced PD by modulating the Bmal1/Nrf2 pathway, which in turn suppressed ferroptosis and promoted mitophagy/autophagy, underscoring the interplay between circadian rhythm regulation and iron homeostasis (Abd El-Khalik et al., 2025).

### 6.3 Activators of the Nrf2–GPX4 antioxidant axis

A substantial body of evidence points to the Nrf2–GPX4 axis as a central node for ferroptosis regulation. Carvedilol, a β-blocker with pleiotropic antioxidant properties, was shown to preserve Nrf2 and GPX4 levels while reducing ACSL4 and NF-κB in a rotenone rat model, leading to improved motor function and dopaminergic neuron survival (Abuelezz et al., 2025). Likewise, DL-3-n-butylphthalide (NBP) reduced iron deposition, upregulated xCT and GPX4, and suppressed oxidative stress in the substantia nigra, correlating with behavioral recovery (Hu et al., 2023). Idebenone, a CoQ10 analog, similarly increased GPX4 and NAD(P)H dehydrogenase [quinone]-1 (NQO1) expression while decreasing lipid peroxidation, suggesting that enhancing mitochondrial electron transport and antioxidant defense are interlinked strategie (Avcı et al., 2020). Hydrogen sulfide (H_2_S) donors (e.g., NaHS) have also been shown to activate the Nrf2 pathway, upregulate GPX4 and FTH1, and inhibit ferroptosis in both hippocampal and dopaminergic neurons (Chen et al., 2025; Shuai et al., 2026). Mechanistically, H_2_S was found to stabilize iron-sulfur clusters and modulate iron efflux via ABCB8, further linking sulfur metabolism to ferroptosis susceptibility (Linjacki et al., 2024).

### 6.4 Modulators of lipid peroxidation and mitochondrial integrity

Given that lipid peroxidation is the executioner of ferroptosis, compounds that directly scavenge lipid radicals or modulate lipid metabolism have shown promise. The oxindole-curcumin hybrid GIF-2165X-G1 and N,N-dimethylaniline derivatives (GIF-2014, GIF-2115) were identified as selective ferroptosis inhibitors that reduced mitochondrial ROS and intracellular Fe^2+^ without affecting apoptosis, demonstrating that ferroptosis and apoptosis can be pharmacologically dissected (Hirata et al., 2022). Idebenone and NBP also reduced malondialdehyde (MDA) levels and preserved mitochondrial ultrastructure, reinforcing the importance of mitochondrial integrity in ferroptosis prevention (Avcı et al., 2020; Hu et al., 2023).

### 6.5 Multi-target kinase inhibitors and HSP90 modulation

Lapatinib and pazopanib, both FDA-approved kinase inhibitors, were repurposed to counteract rotenone-induced PD via ferroptosis and necroptosis inhibition. Lapatinib suppressed the p-EGFR/PLCγ/PKCβII/ACSL4 pathway and restored the NRF2/GPX4/GSH antioxidant system, while also inhibiting the HSP90/CDC37 chaperone complex, which stabilizes multiple PD-relevant proteins including LRRK2 and α-synuclein (Mansour et al., 2023a). Pazopanib similarly inhibited necroptosis (via RIPK1/RIPK3/MLKL) and ferroptosis (via the p-VEGFR2/PKCβII/ACSL4 axis) and reduced iron accumulation and 4-HNE levels, highlighting the potential of multi-target approaches in complex neurodegenerative diseases (Mansour et al., 2023b). These studies illustrate that targeting shared regulatory nodes—such as HSP90 or kinase signaling cascades—can simultaneously suppress multiple cell death pathways.

### 6.6 Emerging targets: gut microbiota and PPAR signaling

Recent work has identified the gut microbiota–CXCL1–PPARγ axis as a novel upstream regulator of ferroptosis and neuroinflammation. In rotenone-fed aged mice, gut dysbiosis led to elevated serum CXCL1, which in turn suppressed PPARγ signaling in the substantia nigra, promoting microglial activation, iron deposition, and ferroptosis (Ma et al., 2024). Remarkably, oral administration of linoleic acid—a PPARγ agonist—blocked these pathological events and rescued dopaminergic neurons, suggesting that targeting peripheral inflammation and lipid signaling may offer a disease-modifying strategy for PD s (Ma et al., 2024).

In summary, the studies reviewed here collectively demonstrate that ferroptosis is a druggable mechanism in rotenone-induced PD models. The common principle across all successful interventions is the restoration of redox homeostasis—either by directly inhibiting lipid peroxidation, chelating iron, enhancing endogenous antioxidant defenses (Nrf2-GPX4), or modulating upstream signaling pathways (e.g., ferritinophagy, kinase cascades, or PPARγ). These findings provide a strong rationale for further translational development of ferroptosis inhibitors as potential disease-modifying therapies for PD.

## 7 Conclusions and perspectives

The strongest evidence from rotenone models supports ferroptosis as a functionally important driver of dopaminergic neurodegeneration. Rotenone consistently disrupts iron homeostasis, inactivates the System Xc^−^–GPX4 axis, promotes ACSL4/LPCAT3-dependent PUFA-phospholipid peroxidation, induces NCOA4-mediated ferritinophagy, amplifies mitochondrial ROS, and engages microglial CR3/NOX2 neuroinflammation. Critically, direct ferroptosis inhibitors such as Fer-1 and liproxstatin-1, iron chelators such as DFO and deferiprone, and ferritinophagy blockade by CNO attenuate rotenone-induced iron accumulation, lipid peroxidation, and dopaminergic loss, providing the most specific evidence for a ferroptotic mechanism. Nrf2/GPX4 activators, mitochondrial agents, and kinase/HSP90 inhibitors also protect, but their effects are multi-pathway and not exclusively ferroptosis-dependent.

Several limitations temper translation. Rotenone is a conditional complex I inhibition model rather than idiopathic PD, and its phenotype varies with dose, route, species, sex, and duration. Most mechanistic evidence remains preclinical, and ferroptosis specificity is often inferred from marker changes rather than demonstrated by rescue with ferroptosis inducers or reversal by selective inhibitors. Biomarkers for patient stratification, target engagement, and pharmacodynamic monitoring remain insufficient, and long-term inhibition of a physiological redox pathway raises safety concerns. Validation in genetic PD models, human iPSC-derived neurons, and idiopathic PD cohorts is therefore essential.

Realistically, the most immediate translational opportunity is mechanism-guided repurposing of safe, brain-penetrant drugs such as carvedilol, idebenone, lapatinib, and pazopanib, coupled with early-phase trials that measure iron, lipid peroxidation, GPX4 activity, and oxidized phospholipid biomarkers. Future strategies should prioritize selective modulation of NCOA4-mediated ferritinophagy, the SIRT1–Nrf2–GPX4 axis, and microglial gp91phox, and should test time-limited combinations only when both ferroptotic and parallel death pathways are active. Thus, ferroptosis is a promising but context-dependent therapeutic target in rotenone-related parkinsonism, and its clinical value will depend on rigorous specificity, biomarker enrichment, and validation beyond rotenone models.
